# Temperate Propolis Has Anti-Inflammatory Effects and Is a Potent Inhibitor of Nitric Oxide Formation in Macrophages

**DOI:** 10.3390/metabo10100413

**Published:** 2020-10-14

**Authors:** Samyah Alanazi, Naif Alenzi, James Fearnley, William Harnett, David G. Watson

**Affiliations:** 1 Clinical Laboratory Sciences Department, College of Applied Medical Sciences, King Saud University, Riyad 11451, Saudi Arabia; saalanazi@KSU.EDU.SA; 2 Research and Laboratories Sector, National Drug and Cosmetic Control Laboratories (NDCCL), Saudi Food and Drug Authority, Riyad 13513, Saudi Arabia; NDenzi@sfda.gov.sa; 3 Apiceutical Research Centre, 6 Hunter Street, Whitby, North Yorkshire YO21 3DA, UK; james.fearnley@beearc.com; 4 Strathclyde Institute of Pharmacy and Biomedical Sciences, University of Strathclyde, 161, Cathedral Street, Glasgow G4 0RE, UK

**Keywords:** propolis, pro- and anti-inflammatory cytokines, LPS stimulation, bone marrow derived macrophages, metabolomics

## Abstract

Previous research has shown that propolis has immunomodulatory activity. Extracts from two UK propolis samples were assessed for their anti-inflammatory activities by investigating their ability to alter the production of the cytokines: tumour necrosis factor-α (TNF-α), interleukin-1β (IL-1β), IL-6, and IL-10 from mouse bone marrow-derived macrophages co-stimulated with lipopolysaccharide (LPS). The propolis extracts suppressed the secretion of IL-1β and IL-6 with less effect on TNFα. In addition, propolis reduced the levels of nitric oxide formed by LPS-stimulated macrophages. Metabolomic profiling was carried out by liquid chromatography (LC) coupled with mass spectrometry (MS) on a ZIC-pHILIC column. LPS increased the levels of intermediates involved in nitric oxide biosynthesis; propolis lowered many of these. In addition, LPS produced an increase in itaconate and citrate, and propolis treatment increased itaconate still further while greatly reducing citrate levels. Moreover, LPS treatment increased levels of glutathione (GSH) and intermediates in its biosynthesis, while propolis treatment boosted these still further. In addition, propolis treatment greatly increased levels of uridine diphosphate (UDP)–sugar conjugates. Overall, the results showed that propolis extracts exert an anti-inflammatory effect by the inhibition of pro-inflammatory cytokines and by the metabolic reprogramming of LPS activity in macrophages.

## 1. Introduction

Propolis is collected by bees from plants surrounding the beehive and mixed with bee saliva and with bees wax. It is used to cover surfaces within the hive prior to laying down the honey comb and to plug gaps within the hive in order to exclude the outside world. In the UK, Northern Europe, and North America, propolis is collected from poplar trees and their relatives; in Southern Europe and North Africa, much of it comes from Cypress trees; and in tropical regions such as Brazil and West Africa, propolis is usually collected from many different plant sources [1,2]. There are numerous literature reports on the immunomodulatory effects of propolis [3,4,5,6,7,8]. In a recent paper, we studied several types of propolis for their effects on the metabolic response of THP-1 cells to treatment with lipopolysaccharide (LPS), and for one sample from Cameroon, which inhibited cytokine release, we studied the metabolomics of the immune response and found a clear effect of the propolis in inhibiting purine nucleotide phosphorylase [8]. In addition, there have been some quite extensive trials of propolis in humans aimed at affecting the immune response [9,10,11,12,13]. The commonly available propolis supplements, such as glycerol-based tinctures, lozenges, and capsules, are mainly based on temperate propolis such as that collected by bees in the UK. Thus, in this paper, we study the effects of two propolis samples collected in the UK on the metabolic response of primary mouse macrophages challenged with LPS.

There have been several comprehensive studies of the metabolomics response of primary macrophages to LPS. The major effects of LPS on the metabolome of macrophages can be summarised as follows: there is a large surge in glycolytic flux with increases in glycolytic intermediates within the cells, an accumulation of intermediates in the tricarboxylic acid (TCA) cycle, and a rise in the intermediates in the pentose phosphate pathway (PPP) [14,15,16,17,18,19,20]. Macrophages display two main phenotypes, M1 and M2. The M1 type is induced by LPS treatment [14,15,16,17,18,19,20] and is responsible for the destruction of pathogens, whereas M2 macrophages are responsible for longer-term resistance to parasites and wound repair [15]. The two phenotypes have different metabolic phenotypes. M1 produces nitric oxide (NO), which acts as a messenger to promote phagocytosis and also acts directly in destroying microbes via the formation of peroxynitrite through the reaction between NO and superoxide. NO production requires increased levels of NADPH, which is required to convert hydroxy arginine to citrulline and NO [21]. NO formation is an important component in macrophage activation. The demand for NADPH is high, since the respiratory burst triggered by stimulation of the macrophages with a pathogen product such as LPS promotes superoxide production, which also consumes large quantities of NADPH. Nevertheless, the respiratory burst is transient and may be turned off by antioxidant enzymes within the cell such as superoxide dismutase [22]. However, this enzyme has GSH as a co-factor, and this results in formation of GSSG, which also requires NADPH to recycle it back to GSH. The main source of NADPH within the cell is the oxidative arm of the pentose phosphate pathway, which converts glucose 6-phosphate into 6-phosphogluconate, producing NADPH in the process. There are other sources of NADPH, but they are more minor and result from the action of isocitrate lyase [16]. It has been proposed that the accumulation of citrate in macrophages following stimulation with LPS is an indication of the diversion of this metabolite into NADPH production [17]. A major recent topic of research has been the alteration of central metabolism when M1 type macrophages are stimulated with LPS. The macrophages accumulate citrate and succinate, since the TCA cycle becomes disrupted after succinate, and there is a switch in metabolism towards glycolysis for ATP generation away from the TCA cycle [17,19,20]. In addition, some of the flux through the TCA cycle is diverted into the antimicrobial metabolite itaconic acid [23]. The M2 phenotype does not produce NO, since arginine is metabolised via arginase to produce ornithine rather than citrulline [17,19,20]. In M2 macrophages, there is shift towards the production of the sugar conjugates required for the formation of glycan chains on proteins such as UDP-*N*-acetyl glucosamine, which is involved in the formation of the pattern recognition receptor CD206, which is strongly expressed in M2-type macrophages [19,20]. M2 macrophages do not switch their metabolism towards glycolysis and remain committed to oxidative phosphorylation and show greater levels of fatty acid metabolism in comparison to M1 macrophages [17,19,20].

Temperate propolis is composed of several hundred components but with around 20 major constituents, which are flavonoids, flavonoid esters, and phenylpropanoid compounds [24]. Flavonoids have been shown to inhibit nitric oxide synthase in macrophages and to bind to the PPAR-γ receptor [25]. Thus, it might be expected at the outset that propolis treatment would affect NO biosynthesis and fatty acid metabolism. The aim of the current study was to study the effect of two samples of temperate propolis on the metabolomic changes induced in primary macrophages by treatment with LPS.

## 2. Results

### 2.1. The Effect of Propolis on NO Production and Cytokine Production by Macrophages

**Figure 1:**
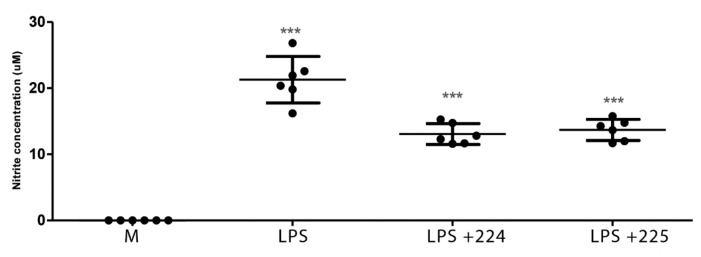
Effect of two samples of propolis (50 µg/mL) from the UK on nitric oxide (NO) production by macrophages stimulated by lipopolysaccharide (LPS); *n* = 6 *** = *p* value < 0.001. Comparisons of LPS against medium and LPS + propolis samples 224 and 225 against LPS. Medium alone = M.

**Figure 2:**
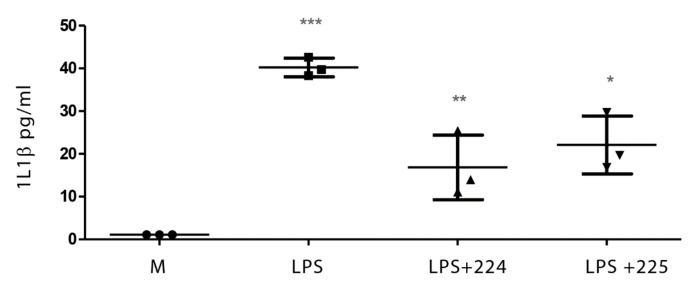
Effect of UK propolis (50 µg/mL) on interleukin-1β (IL-1β) production by LPS-stimulated macrophages; *n* = 3 * *p* value < 0.05, ** *p* value < 0.01, and *** *p* value < 0.001. Comparisons of LPS against medium and LPS + propolis samples 224 and 225 against LPS. Medium alone = M.

**Figure 3:**
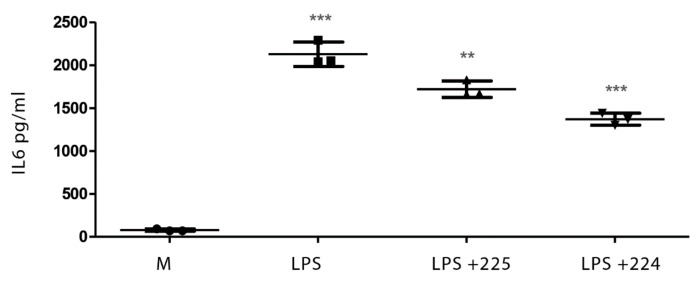
The effect of UK propolis on IL-6 production in LPS-stimulated macrophages; *n* = 3 ** *p* value < 0.01, *** *p* value < 0.001. Comparisons of LPS against medium and LPS + propolis samples 224 and 225 against LPS. Medium alone = M.

**Figure 4:**
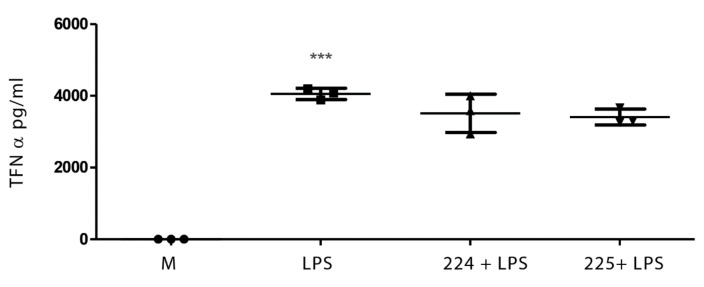
The effect of UK propolis on tumour necrosis factor-α (TNF-α) production in LPS-stimulated macrophages; *n* = 3 *** *p* value < 0.001. Comparisons of LPS against medium and LPS + propolis samples 224 and 225 against LPS.

**Figure 5:**
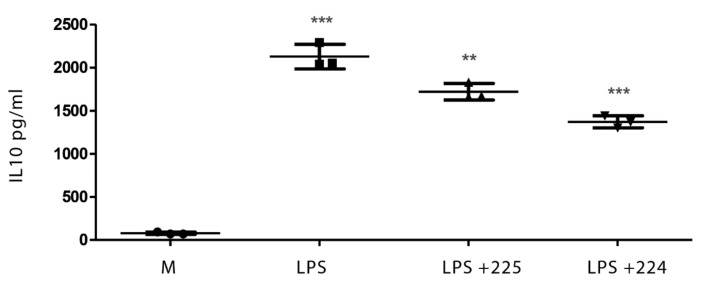
The effect of UK propolis on IL-10 production in LPS-stimulated macrophages; *n* = 3 ** *p* value < 0.01 *** *p* value < 0.001. Comparisons of LPS against medium and LPS + propolis samples 224 and 225 against LPS.

Figure 1 shows the effect of LPS on NO production in LPS-activated macrophages and the effect of two different types of propolis from the UK (from Essex and from the Midlands) in inhibiting NO production. Similarly, treatment with LPS elevated the levels of interleukin-1β (IL-1β), tumour necrosis factor-α (TFN-α), and IL-6 in macrophages (Figure 2, Figure 3 and Figure 4), whereas the propolis samples clearly lowered IL-1β and IL-6 but with less clear effects in lowering TFN-α. Propolis treatment lowers the production of IL-10, which is considered to be an anti-inflammatory cytokine (Figure 5). This might imply that the propolis does have some pro-inflammatory effects. Propolis is regarded as being immunomodulatory, and it is conceivable that it might both regulate and enhance the immune response. The regulation of IL-10 levels in immune cells is complex [26] and is regulated by other cytokines such as interferons, which might also be regulated by the propolis.

### 2.2. Propolis Does Not Affect Cell Viability

**Figure 6:**
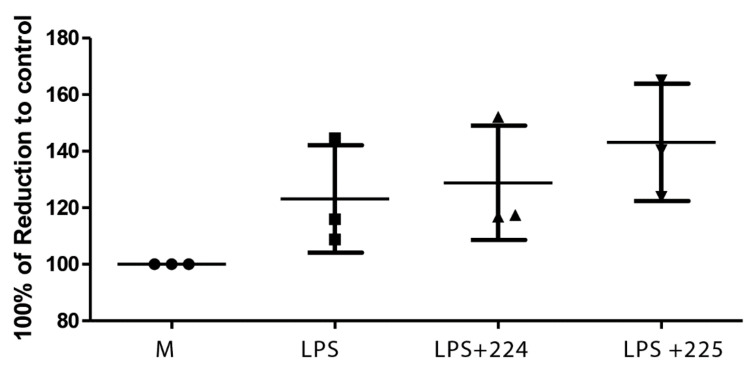
The effect of UK propolis on the viability of LPS-stimulated macrophages measured by using an Alamar blue assay [8].

The propolis samples did not affect cell viability of the macrophages at the concentration used (Figure 6).

### 2.3. The Effect of Propolis on the Metabolomic Profile of Macrophages

Table 1 lists the numerous significant effects on cellular metabolites of the two propolis samples on the response of the macrophages to LPS treatment.

#### 2.3.1. Propolis Treatment Inhibits NO Formation

**Figure 7:**
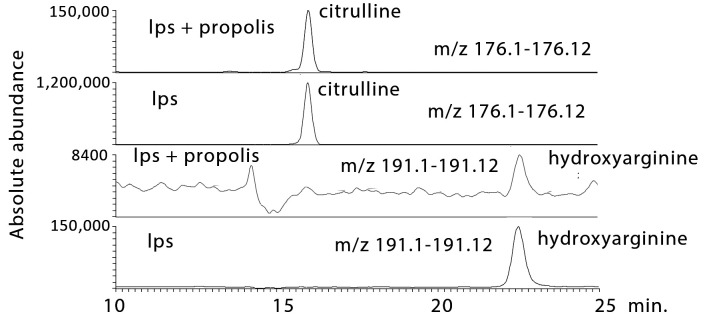
Extracted ion traces showing decreases in hydroxy arginine and citrulline in LPS-stimulated macrophages treated with propolis.

Analysis of the metabolomics data shows the clear effects of LPS in greatly elevating hydroxyarginine, the intermediate in NO production from arginine, and citrulline, the product remaining after NO formation. The UK propolis samples have a marked effect on lowering both hydroxyarginine and citrulline (Figure 7), which fits with the direct measurement indicating lowered NO formation (Figure 1). The propolis samples also increase arginosuccinate, which is involved in recycling citrulline back to arginine in order to make more NO, and the accumulation could be due to the inhibition of conversion of this substrate into arginine. There were no direct effects on arginine levels, which were the same in the macrophages treated with LPS and LPS + propolis.

#### 2.3.2. Propolis Treatment May Promote Energy Metabolism and Stimulate the Formation of High Energy Phosphates

**Figure 8:**
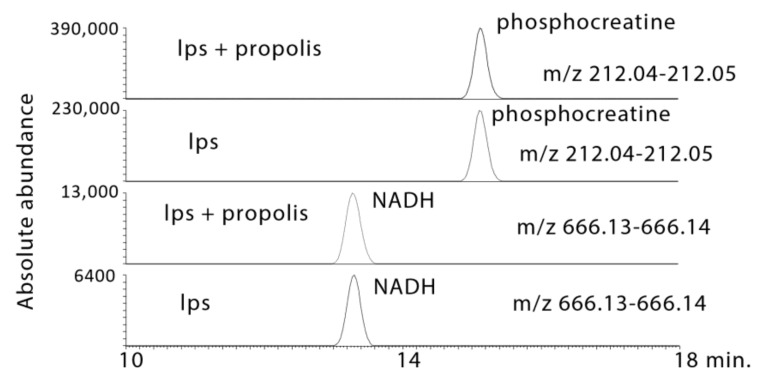
Extracted ion traces showing increased in phosphocreatine and NADH in LPS-stimulated macrophages treated with propolis.

**Figure 9:**
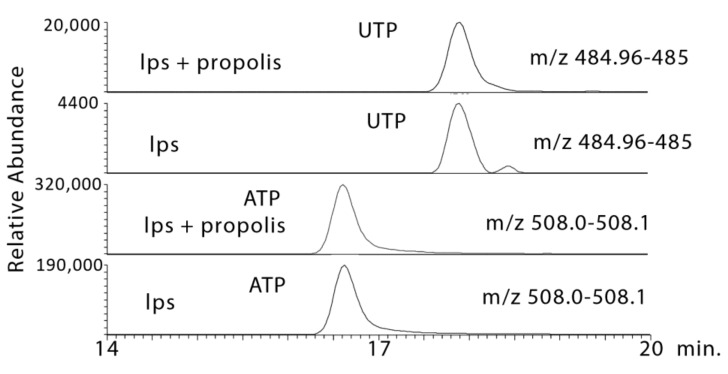
Extracted ion traces showing increases in UTP and ATP in LPS-stimulated macrophages treated with propolis.

LPS produced moderate increases in NADH and ATP, which are largely derived from the TCA cycle or fatty acid oxidation, and propolis treatment increased NADH and ATP levels further (Figure 8 and Figure 9). In addition, the levels of other high energy phosphates such as creatine phosphate and uridine triphosphate (UTP) were increased by propolis treatment (Figure 8 and Figure 9). LPS treatment had a marked effect on some glycolysis intermediates with a large increase in glyceraldehyde 3-phosphate (G3P), which was increased still further by the propolis treatments (Table 1).

#### 2.3.3. Propolis Treatment Stimulates Formation of Amino Sugars

**Figure 10:**
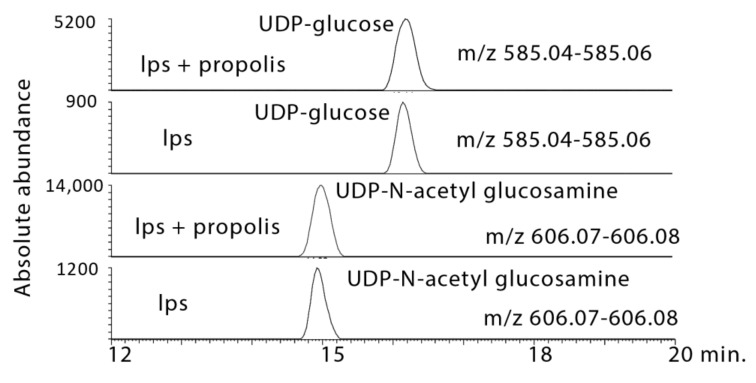
Extracted ion trace showing increases in UDP–glucose and UDP–*N*-acetyl glucosamine in LPS-stimulated macrophages treated with propolis.

The propolis treatments caused increases in the levels of the amino sugars *N*-acetyl glucosamine, neuraminate, and *N*-acetylglucosamine phosphate (Table 1) as well as increases in UDP–*N*-acetyl glucosamine and UDP–glucose (Figure 10). All these intermediates are increased in the LPS-treated samples and markedly increased in the propolis-treated samples.

#### 2.3.4. Propolis Treatment Lowers Citrate and Increases Itaconate

**Figure 11:**
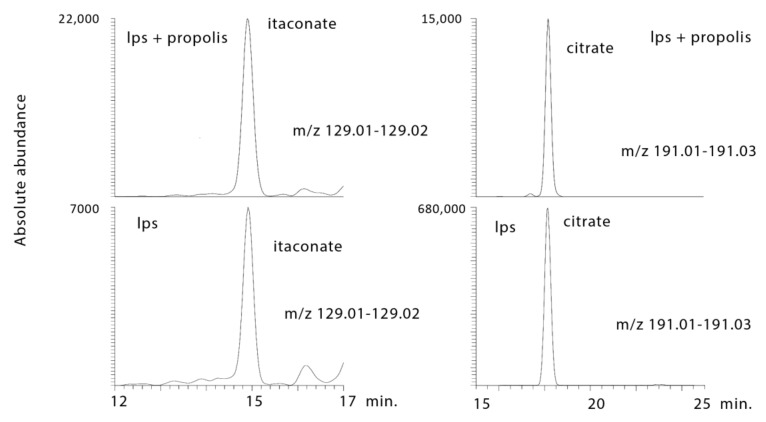
Extracted ion traces showing a decrease in citrate and increase in itaconic acid in LPS-stimulated macrophages treated with propolis.

Citrate was markedly lowered by propolis treatment in comparison with LPS treatment alone, and the anti-inflammatory compound itaconic acid was markedly increased by propolis treatment (Figure 11).

### 2.4. Propolis Treatment Increases GSH Levels

**Figure 12:**
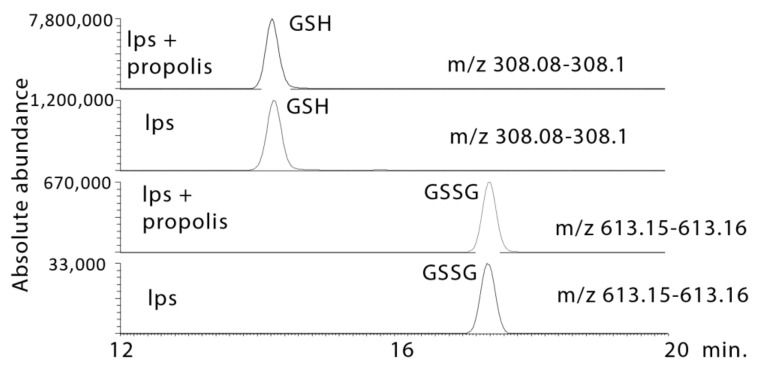
Extracted ion traces showing increased in GSH and oxidised glutathione (GSSG) in LPS-stimulated macrophages treated with propolis.

Another important pathway in macrophages is glutathione metabolism, since GSH may modulate the response of the macrophages and may be responsible for damping down the respiratory burst following stimulation with a pathogen product [17,19,20]. The propolis treatments increase GSH levels, and the level of its oxidation product GSSG is also elevated greatly in comparison to LPS treatment alone (Figure 12). In addition, the propolis treatments elevate all the intermediates involved in GSH biosynthesis: glycine, cysteine, glutamate, and gammaglutamyl cysteine (Table 1).

### 2.5. Propolis Treatment Increases Fatty Acid Metabolism

One of the major effects of the propolis treatment is on the levels of certain fatty acids, which are markedly increased, suggesting that this might be due to triglyceride hydrolysis and a switch towards fatty acid metabolism (Table 1). The increase in fatty acid metabolism is underlined by the elevation of several acyl carnitines, which are required for the transport of fatty acids into mitochondria so that β-oxidation can be carried out. A signature of M2 macrophages in comparison with M1 macrophages is increased fatty acid metabolism [17,19,20]. Some long-chain fatty acids are increased by LPS treatment but are markedly lowered by the propolis treatments, including eicosatetraencoic acid, which, as the precursor of prostaglandins, can be considered pro-inflammatory.

## 3. Discussion

It is clear from the viability data and the enhancement of energy metabolites such as ATP and creatine phosphate that propolis if anything increases the viability of the cultured macrophages. The cytokine assays largely support the reports [3,4,5,6] that propolis is anti-inflammatory and the metabolomics data almost perfectly support the idea that propolis treatment is pushing the macrophages towards an M2-like character. The conversion of arginine to citrulline with the formation of NO is inhibited, and levels of GSH and GSSH are increased, pointing towards the promotion of activity against ROS generation; a number of intermediates required for GSH biosynthesis are elevated by propolis treatment. UDP-*N*-acetyl glucosamine, which is used for glycoprotein formation in M2-type macrophages, is elevated, as are intermediates required for its biosynthesis, UTP, *N*-acetylglucosamine, and *N*-acetylglucosamine phosphate. Of note, UDP-*N*-acetyl glucosamine is required as a substrate for the production of glycans on proteins such as the CD206 receptor, which is abundantly expressed in M2 macrophages. It is well established that in M2 macrophages, some of the glycolytic flux is diverted into the amino sugar pathway [20]. The propolis-treated macrophages accumulate glyceraldehyde phosphate to an even greater extent than macrophages treated with LPS alone, suggesting the inhibition of glyceraldehyde phosphate dehydrogenase; this process can promote methylglyoxal formation from glyceraldehyde phosphate and dihydroxy acetone phosphate [27]. The elevation of methylglyoxal by the propolis treatments is supported by a large increase in lactoyl glutathione, which is the cellular detoxification product of methyl glyoxal [28]. Methyl glyoxal can react with arginine residues in proteins generating the advanced glycation product carboxyethylarginine, which is elevated following LPS treatment and further elevated by propolis treatment. Carboxyethyl arginine has been shown to be an inhibitor of NO production [29]. Citrate accumulates in M1-type macrophages and is important in sustaining the inflammatory response through supporting fatty acid biosynthesis [17,19,20,30] and the production of ROS. The propolis treatments hugely deplete citrate. LPS treatment increases the levels of the anti-inflammatory compound itaconic acid, and propolis treatment increases its levels still further; there is some evidence that itaconate stimulates M2 macrophage polarisation [23].

The metabolism of eicosatetranenoic acid is carried out by peroxisomes [31], and it has been shown that PPAR-γ receptor activation can affect macrophage polarisation [32]. Flavonoids have been shown to inhibit inducible cycloxgenase synthase and nitric oxide synthase by binding to the PPAR-γ receptor in macrophages [25]. Thus, it would seem that part of the action of propolis may be due to promoting PPAR-γ receptor activation. This has been found to be a feature of many natural products [33], including flavonoids that are present in the propolis extracts. It has been observed that fatty acid metabolism is promoted over glycolysis in M2 macrophages in comparison with M1 macrophages, and the presence of increased levels of several hydroxylated long-chain carnitines and greatly elevated levels of partially oxidised fatty acids in the propolis-treated samples suggest that the propolis treatments are promoting the M2 character in the macrophages [34,35].

The two samples used in this study were typical samples of temperate propolis collected from poplar buds where the composition is well established [24,36]. There are some variations between samples 224 and 225. The flavanones pinocembrin and pinobanksin (Figures S2 and S4) appear to be qualitatively similar in the samples, whereas the flavone chrysin and the flavonols galangin methyl ethers and kaempferol appear to be markedly higher in sample 224 (Figures S1, S3, S5 and S6). It was previously observed that flavones and flavonols had a greater effect in inhibiting nitric oxide production by macrophages than flavanones [37,38,39]. This might explain some of the difference in effect between 224 and 225 on the macrophages.

## 4. Materials and Methods

### 4.1. Chemicals and Reagents

Fetal bovine serum, DMEM (1X), DMEM phenol red free, and RPM-1640 medium were obtained from Gibco. Glutamine solution, PBS, and penicillin/streptomycin solution were obtained from Lonza. Six-well cell culture plates were purchased from Thermo Fisher Scientific; 96-well cell culture plates were purchased from TPP, Switzerland. Ethanol, trypan blue stain, bovine serum albumin, EDTA, carbonyl cyanide m-chlorophenyl hydrazine, oligomycin A, and ammonium carbonate were obtained from Sigma-Aldrich, Dorset, UK. HPLC grade methanol, acetonitrile, and water were obtained from Fisher Scientific, Leicestershire, UK.

### 4.2. Preparation of Propolis Extracts

The two UK propolis samples were obtained by James Fearnley; sample 224 was from Essex and sample 225 was from the Midlands. Ethanol extracts of approximately 10 g propolis were prepared by vigorous mixing and sonication for 60 min using a sonicating bath (Fisher Scientific, Loughborough, UK). The extracts were filtered and the propolis was re-extracted twice with 100 mL of ethanol (Fisher Scientific, Loughborough, UK). The extracts were combined and evaporated, and the residue was stored at room temperature until required for the assays.

### 4.3. Generation of Bone Marrow-Derived Macrophages (BMMs)

All experiments were approved by and conducted in accordance with the Animal Welfare and Ethical Review Board of the University of Strathclyde and UK Home Office Regulations. Bone marrow was collected from the femur and tibia bones of 6–8-week-old male or female BALB/c mice, which were bred at Strathclyde University and killed by cervical dislocation. Then, bones were dissected from adherent tissues and washed briefly with 70% ethanol. In sterile conditions, under a tissue culture hood, the bone ends were cut to allow bone marrow elution through washing the bone DMEM medium. Then, the eluted bone marrow was collected, filtered using a cell strainer, and centrifuged at 400× *g* for 5 min. The supernatants were next aspirated and replaced with a known amount of fresh complete DMEM medium to count the obtained cells, using trypan blue stain in order to culture them at the required density. Then, cells were plated and cultured on tissue culture Petri dishes at a density of 2 × 10^6^ cells/mL in complete DMEM with 20% L929 cell supernatant [40] and maintained at 37 °C in a humidified atmosphere of 5% (*v*/*v*) CO_2_. Fresh complete DMEM supplemented with 20% L929 cell supernatant was added on day 4 to feed the macrophages. On day 7, the cells were harvested by scraping them into 5 mL complete DMEM at 4 °C to allow adherent cell detachment, and they were then collected for further centrifugation at 400× *g* for 5 min. Then, the viability and number of cells was checked using trypan blue stain followed by identification by flow cytometry and plating according to the desired experiments.

### 4.4. Flow Cytometry

Re-suspended cells with a density of 0.5 × 10^6^/ fluorescence activated cell sorting (FACS) tube were incubated with anti-mouse CD16/CD32 for 5 min to block subsequent nonspecific binding of antibodies to Fc receptors. Cells were next incubated with antibodies specific for CD11b-APC (BD Pharmingen) and F4/80-FITC (eBioscience) along with the Fluorescence Minus One (FMO) controls (eBioscience and BD Pharmingen) and placed at 4 °C for 25 min, after which they were washed in FACS buffer (2% Bovine Serum Albumin (Sigma-Aldrich, Poole, Dorset, UK) in PBS (Lonza, Slough, UK) with 2 mM EDTA). Then, the cells were re-suspended in FACS buffer to render them ready for flow cytometry analysis. Flow cytometry was carried out using a FACS Canto instrument (BD Pharmingen, San Jose, CA, USA). The purity of differentiated bone marrow-derived macrophages was determined by the measurement of CD11b and F4/-80 double positive cells. Cultures of >90% were used for cytokine and metabolomics assays.

### 4.5. Effect of Propolis Treatment on LPS-Activated Macrophages

The BMMs were plated at a concentration of 2 × 10^6^ cells/2 mL of complete RPMI medium (RPMI-1640 (Lonza), 2 mM glutamine (Lonza), 50 U/mL penicillin (Lonza), 50 µg/mL streptomycin (Lonza), 10% FCS (Gibco, Paisley, UK)) in 6-well plates, with 5 to 6 replicates/each condition used, and then rested for 5 h or overnight. To study the effect of adding propolis on the macrophage metabolome, the two sample extracts were added at a concentration of 50 µg/mL along with an equivalent amount of medium only added to the control group. Then, treated BMMs were incubated for 18 h at 37 °C in a humidified atmosphere of 5% (*v*/*v*) CO_2_. After 18 h of incubation, LPS (*Escherichia coli*, Sigma Aldrich) was added to give a concentration of 100 ng/mL, and incubation was continued for a further 24 h.

Cell extracts were prepared by washing the cells once with warm PBS before harvesting the cells in a chilled extraction solution (MeOH/MeCN/H_2_O, 50:30:20 *v*/*v*) with a concentration of 1 mL of extraction mix per 2 × 10^6^ cells. Then, cell lysates were collected and shaken at 1200 rpm for 20 min at 4 °C before being centrifuged at 0 °C at 13,000 rpm for 15 min. Then, the supernatants were collected and transferred into auto sampler vials for loading into the LC-MS autosampler or storage at −80 °C until analysis.

### 4.6. Measurement of Cell Viability

The BMM cells were seeded at a density of 2 × 10^5^ cells/well in 96-well plates and incubated for 24 h at 37 °C in a humidified atmosphere of 5% CO_2_. After 4 h of resting, to allow adhesion, the cells were treated with medium only for the negative control cells and propolis extract (50 µg/mL) and all incubated for a further 24 h. After 24 h, LPS was added to all cells except the control incubations, for which incubation was carried out for 24 h. Four hours before the end of the incubation, Alamar blue [8] was added to all cells, with a slight shaking, and then the plates were returned to the incubator until the end of treatment. To read the cell viability, 100 µL of all conditions was pipetted into new plates to measure fluorescence at excitation: 530 nm, emission: 590 nm using a Polarstar Omega plate reader (BMG Labtech, Aylesbury, UK).

### 4.7. Measurement of NO Production in BMMs

BMMs were plated and stimulated with propolis pre-treatment followed by LPS activation. First, 50 μL aliquots of cell supernatant were collected and added into wells of a 96-well plate. Then, Griess reagents (A + B) were mixed in a ratio of 1:1 [2% (*w*/*v*) sulphanilamide in 5% (*v*/*v*) H_3_PO_4_ and 0.2% (*w*/*v*) naphylethylenediamine HCl in water], and 50 μL of the mix were added to the cell supernatants in each well. Then, the 96-well plate was incubated in the dark for 10 min. Then, the absorbance was read using a Polarstar Omega plate reader at 540 nm. Nitrite production was determined relative to a standard curve constructed with solutions of sodium nitrite (NaNO_2_) as described previously [41] from a 10 mM stock solution of NaNO_2_ prepared in complete RPMI 1640 cell medium.

### 4.8. Cytokine Assays

BMMs were plated and stimulated with propolis pre-treatment followed by LPS activation. First, 50 μL of cell supernatant were collected for analysis using ELISA Ready-Set-Go kits purchased from Thermo Fisher Scientific (Loughborough, UK).

The assays were performed according to the manufacturer’s instructions to quantify the release of cytokines: (TNF-α, IL-1β, IL-6, and IL-10). The reaction was stopped using 2 N sulphuric acid. The plates were read using a SpectraMax M5 plate reader (Molecular Devices, Sunnyvale, CA, USA) at 560 nm, and the absorbance values were corrected by subtracting readings. The data obtained were analysed using Gen5 and Prism 7.

### 4.9. Liquid Chromatography/Mass Spectroscopy (LC/MS)

The chromatographic conditions were set as follows: A ZICpHILIC column (150 × 4.6 mm × 5 µm, Hichrom, Reading, UK) was eluted with a linear gradient over 30 min between 20 mM ammonium carbonate (pH 9.2)/MeCN (20:80) at 0 min and 20 mM ammonium carbonate (pH 9.2)/MeCN (20:80) at 30 min with a flow rate of 0.3 mL/min, followed by washing with 20 mM ammonium carbonate MeCN (95:5) for 5 min and then re-equilibration with the starting conditions for 10 min. LC/MS was carried out by using a Dionex 3000 HPLC pump coupled to an Exactive (Orbitrap) mass spectrometer from Thermo Fisher Scientific (Bremen, Germany). The spray voltage was 4.5 kV for positive mode and 4.0 kV for negative mode. The temperature of the ion transfer capillary was 275 °C and sheath and auxiliary gas were 50 and 17 arbitrary units, respectively. The full scan range was 75 to 1200 *m*/*z* for both positive and negative modes. The data were recorded using the Xcalibur 2.1.0 software package (Thermo Fisher Scientific, Hemel Hempstead, UK). The signals of 83.0604 *m*/*z* (2 × ACN + H) and 91.0037 *m*/*z* (2 × formate-H) were selected as lock masses for the positive and negative modes, respectively, during each analytical run.

### 4.10. Metabolomic Data Analysis

Raw data from untargeted metabolomic studies were putatively identified and processed using Mzmine [42] Prior to further analysis, data were filtered in which metabolites of low intensities (<1000 peak height) and metabolites that did not show any significant fold changes were excluded in order to simplify the data for interpretation. Accurate masses were searched against an in-house database, and in addition, retention times for 200 metabolites were matched against standard mixtures run at the same time as the samples [43]. Otherwise, metabolite matches were based on accurate masses < 3 ppm and identified to MSI levels 1, where the retention time matched that of a standard or to level 2 [44]. Details of the metabolite mixtures were described previously [43].

### 4.11. Statistical Analysis

The extracted data were exported to Excel in order to calculate *p* values and metabolite ratios. Further analysis of the extracted data was carried out by using Metaboanalyst 4.0 [45] in order to determine ANOVA values between the different treatments. A PDF file with mean extracted peak areas and standard error of the mean (SEM) values is provided in Supplementary Materials Appendix 1.

## 5. Conclusions

One might ask what benefit immunomodulation might offer to the bees that collect it. Perhaps some clue to this is from the recent paper indicating that the honey bee microbiome is stabilised by propolis [46], and this could in part be mediated by modulation of the immune response as well as by the control of pathogens by the propolis. Propolis contains hundreds of components with flavonoids and phenylpropanoid compounds being the major components. However, it may act in the form of a complex with more minor components also playing a role in its overall activity. Generally, the well-established anti-protozoal activity of propolis [47] may depend on the activity of the whole mixture, since usually, isolated components are less active or not more active than the extract mixture. The current paper provides strong support for propolis being effective as an anti-inflammatory complex. Propolis has been studied as an immunomodulatory agent in a number of sizeable trials, and its safety in high doses is good [9,10,11,12,13]. There had been no studies examining whether or not the components in propolis are absorbed. However, recently, we carried out a small-scale trial looking at the absorption of the flavonoids from propolis from an oral dose, and it was clear that the flavonoid components within propolis were generally well absorbed [48]. Such variations in effect vs. composition across different samples could provide the basis for a subsequent study. The effects of propolis on the immune response are very interesting, and these in vitro results support the in vivo trials [10,11,12,13] aimed at producing immune modulation. There is a particularly strong need for a more comprehensive study of the absorption and metabolism of propolis components to support any claims with regard to its efficacy in providing immune support.

## Figures and Tables

**Table 1:** Metabolites in LPS-stimulated macrophages that are significantly affected by propolis. §—significantly different between all six treatments (LPS-M, LPS-224, LPS 225, 224-M, 225-M, and 224–225); ¥—Propolis treatments significantly different from controls but not each other; €—Propolis treatments different from LPS but not the control; #—No difference between 224 and LPS but LPS and 225 differ and treatments differ from control. The false discovery rate statistic (FDR) indicates all *p* values < 0.05 are significant. * Matches retention time of standard. *p* = *p* value (*n* = 6 for each treatment). P LM = *p* value LPS vs. medium treatment; P224L = *p* value for propolis sample 224 + LPS vs. LPS; P225L= *p* value propolis sample 225 + LPS vs. LPS. L/M = LPS treated over medium-treated control, 224/L = propolis sample 224 + LPS over LPS alone, 225/L = propolis sample 225+ LPS over LPS alone.

Anova Tukey’s HSD	*m*/*z*	Rt min	*Metabolite*	P LM	L/M	P224L	224/L	P225L	225/L
**Nitric oxide biosynthesis**									
4.8 × 10^−14^ ¥	176.103	16.0	l-Citrulline *	0.001	31.765	0.002	0.199	0.009	0.432
1.8 × 10^−18^ ¥	191.114	22.5	*N*-(Omega)-Hydroxyarginine	0.001	18030.784	0.002	0.056	0.002	0.157
1.0 × 10^−14^ ¥	247.140	14.3	*N*2-(d-1-Carboxyethyl)-l-Arginine	0.002	4.929	<0.001	6.081	<0.001	10.833
3.3 × 10^−15^ ¥	291.129	16.8	*N*-(l-Arginino) Succinate	0.002	24.687	0.002	3.091	0.001	5.223
**Glycolysis/TCA cycle**									
1.9 × 10^−8^ ¥	129.018	14.9	Itaconic Acid *	0.000	125.289	0.002	4.069	0.001	5.428
8.0 × 10^−8^ ¥	168.990	16.0	Glyceraldehyde 3-Phosphate *	0.003	150.353	0.001	10.092	0.001	12.626
1.3 × 10^−7^ ¥	173.021	15.7	Sn-Glycerol 3-Phosphate *	0.002	12.756	0.005	0.289	0.006	0.331
1.4 × 10^−6^ ¥	179.0552	14.7	d-Glucose *	<0.001	0.429	0.002	0.268	<0.001	0.094
3.0 × 10^−16^ ¥	191.0189	18.1	Citrate *	<0.001	>1000	<0.001	0.024	<0.001	0.017
3.7 × 10^−9^ ¥	666.131	13.3	NADH *	0.009	2.221	0.001	2.477	0.001	3.351
6.1 × 10^−7^ #	810.131	12.3	Acetyl-CoA	0.004	4.363	0.480	1.213	<0.001	2.257
**Oxidative stress/glutathione metabolism**									
1.3 × 10^−13^ ¥	76.040	15.9	Glycine *	0.005	2.367	<0.001	7.222	<0.001	8.452
6.5 × 10^−12^ ¥	122.027	14.1	l-Cysteine *	0.027	20.174	0.001	14.423	<0.001	26.437
4.8 × 10^−9^ ¥	241.031	16.1	l-Cystine *	0.009	0.492	0.005	0.108	0.005	0.159
6.1 × 10^−17^ ¥	251.069	14.1	Gamma-l-Glutamyl-l-Cysteine	0.001	14.204	<0.001	8.063	0.001	14.927
6.4 × 10^−11^ ¥	148.060	14.6	l-Glutamate *	0.012	0.729	<0.001	3.126	<0.001	4.074
1.8 × 10^−16^ ¥	308.090	14.2	Glutathione *	0.000	3.903	0.001	6.268	<0.001	8.205
1.6 × 10^−11^ ¥	336.087	14.6	S-Formylglutathione	<0.001	4.273	<0.001	0.536	<0.001	0.518
5.3 × 10^−15^ ¥	338.0488	17.3	S-Sulfanylglutathione	0.080	>1000	0.006	326.271	0.005	320.140
1.8 × 10^−22^ ¥	380.111	12.1	S-Lactoylglutathione *	0.001	>1000	<0.001	7.408	<0.001	10.455
2.4 × 10^−18^ ¥	613.159	17.4	Glutathione Disulphide *	0.001	5.952	<0.001	24.600	<0.001	20.853
**High-energy phosphate metabolism**									
1.1 × 10^−10^ ¥	212.043	15.1	Phosphocreatine *	0.001	2.157	0.006	1.768	0.002	2.739
4.5 × 10^−10^ ¥	348.070	16.6	AMP *	0.035	4.217	0.007	3.455	0.007	5.228
1.8 × 10^−10^ ¥	322.0446	16.0	CMP *	0.002	1.795	0.001	2.107	<0.001	2.095
1.9 × 10^−11^ ¥	489.114	15.3	CDP–Choline *	0.001	22.575	0.001	0.142	0.001	0.191
9.6 × 10^−13^ ¥	484.975	17.9	UTP *	0.001	0.534	<0.001	7.534	<0.001	11.550
8.2 × 10^−11^ ¥	508.002	16.6	ATP *	0.002	1.987	0.001	2.006	0.001	2.416
5.7 × 10^−10^ ¥	523.997	19.3	GTP *	<0.001	7.584	0.060	1.485	0.002	2.214
4.8 × 10^−10^ §	565.0477	16.1	UDP–Glucose *	0.897	1.058	0.002	24.657	0.007	50.760
8.4 × 10^−10^ ¥	606.0744	15.0	UDP–*N*-Acetyl-d-glucosamine *	0.107	3.312	0.008	33.266	0.016	46.857
**Fatty acid metabolism**									
3.5 × 10^−9^ §	185.117	3.9	10-Oxodecanoate	0.318	0.702	<0.001	100.192	0.001	90.767
3.0 × 10^−9^ ¥	204.123	11.1	O-Acetylcarnitine *	<0.001	4.658	<0.001	0.002	0.490	0.002
1.7 × 10^−7^ €	218.138	9.9	O-Propanoylcarnitine	<0.001	0.321	<0.001	2.871	<0.001	2.967
1.9 × 10^−9^ §	227.201	4.2	Tetradecanoic Acid	<0.001	2.432	0.004	1.413	<0.001	1.464
9.2 × 10^−10^ §	225.186	4.2	Tetradecenoic Acid	0.126	2.130	0.025	2.131	<0.001	5.490
3.5 × 10^−14^ §	269.212	4.2	Oxo-Hexadecanoic Acid	0.323	1.305	0.003	38.013	<0.001	257.413
3.4 × 10^−10^ §	271.228	3.8	Hydroxypalmitate	0.193	0.610	<0.001	129.447	<0.001	132.527
1.7 × 10^−9^ §	279.231	4.0	Linoleate	0.350	0.340	<0.001	20.934	<0.001	22.371
4.1 × 10^−8^ §	283.264	3.8	Octadecanoic Acid	<0.001	1.779	<0.001	0.589	<0.001	0.537
1.7 × 10^−9^ §	295.227	3.8	Hydroxyoctadecadienoic Acid	0.293	1.227	0.008	41.194	<0.001	102.576
1.4 × 10^−7^ §	299.259	3.9	Hydroxyoctadecanoic Acid	0.829	0.937	0.020	29.732	<0.001	68.922
1.7 × 10^−6^ §	301.217	4.0	Eicosapentaenoic Acid	0.001	1.822	0.001	0.585	0.001	0.583
1.7 × 10^−10^ §	303.233	4.0	Eicosatetraenoic Acid	0.267	0.938	<0.001	0.511	<0.001	0.509
3.8 × 10^−8^ §	309.280	3.7	Eicosenoic Acid	<0.001	3.417	0.006	0.699	0.001	0.660
1.2 × 10^−9^ §	327.233	3.7	Docosahexaenoic Acid	0.001	1.905	<0.001	0.336	<0.001	0.395
7.8 × 10^−9^ §	333.280	3.7	Docosatrienoic Acid	<0.001	2.118	<0.001	0.485	<0.001	0.468
2.2 × 10^−8^ #	386.289	4.9	Hydroxytetradecenoylcarnitine	0.002	9.282	0.107	1.459	<0.001	6.815
7.1 × 10^−9^ §	414.320	4.9	Hydroxyhexadecenoylcarnitine	0.002	29.177	0.014	2.715	<0.001	4.851
4.0 × 10^−10^ §	428.373	4.6	Stearoylcarnitine	0.005	1.898	0.001	2.351	0.003	1.699
4.3 × 10^−22^ §	442.352	7.6	Hydroxyoctadecenoylcarnitine	0.001	>1000	<0.001	13.029	<0.001	11.175
**Phospholipid biosynthesis**									
1.4 × 10^−3^ €	104.107	14.5	Choline *	<0.001	4.826	<0.001	0.407	<0.001	0.379
8.8 × 10^−13^ ¥	184.073	15.0	Choline Phosphate *	0.001	2.530	<0.001	2.827	0.001	2.582
2.0 × 10^−9^ ¥	258.110	14.5	Sn-Glycero-3-Phosphocholine *	<0.001	5.060	0.001	0.427	0.001	0.400
1.9 × 10^−11^ ¥	568.339	4.6	LPC 22:6	0.009	2.501	<0.001	3.014	<0.001	2.920
5.0 × 10^−8^ €	810.526	3.8	PS 38:5	<0.001	3.432	<0.001	0.186	<0.001	0.291
6.0 × 10^−5^ ¥	820.619	4.1	PC40:5	0.034	1.269	0.002	0.618	0.001	0.572
**Purines and pyrimidines**									
1.1 × 10^−13^ §	136.062	9.7	Adenine *	<0.001	4.250	<0.001	1.708	<0.001	2.160
1.5 × 10^−10^ §	137.046	8.3	Hypoxanthine *	0.002	2.386	0.002	4.181	<0.001	6.198
3.4 × 10^−8^ ¥	243.062	12.0	Uridine *	0.624	1.141	<0.001	3.804	<0.001	4.458
8.6 × 10^−11^ §	251.0784	9.6	Deoxyinosine	0.072	2.383	0.007	21.680	<0.001	35.912
5.3 × 10^−10^ §	284.098	12.7	Guanosine *	0.046	13.235	0.001	23.082	<0.001	29.869
8.3 × 10^−09^ ¥	384.114	14.4	Succinyladenosine	0.050	>1000	0.006	3.782	0.006	2.794
**Aminosugars**									
1.5 × 10^−7^ €	180.086	18.1	d-Glucosamine *	<0.001	6.336	<0.001	0.210	<0.001	0.148
1.2 × 10^−9^ ¥	222.098	12.7	*N*-Acetyl-d-Glucosamine *	0.018	21.617	<0.001	5.012	0.003	6.840
4.6 × 10^−14^ §	266.0895	11.2	Neuraminic Acid	0.164	2.320	<0.001	62.644	<0.001	97.751
8.8 × 10^−15^ ¥	300.0489	15.0	*N*-Acetyl-d-Glucosamine 6-Phosphate *	0.003	0.428	<0.001	27.336	<0.001	26.704
3.2 × 10^−6^ §	310.113	13.2	*N*-Acetylneuraminate	0.013	2.197	0.002	0.170	0.004	0.297

## Supplementary Materials

The following are available online at https://www.mdpi.com/2218-1989/10/10/413/s1, Figure S1: Extracted ion traces for chrysin in samples 225 and 224, Figure S2: Extracted ion traces for pinocembrin in samples 225 and 224, Figure S3: Extracted ion traces for galangin in samples 225 and 224, Figure S4: Extracted ion traces for pinobanksin in samples 225 and 224, Figure S5: Extracted ion traces for galangin methyl ethers in samples 225 and 224, Figure S6: Extracted ion traces for kaempferol in samples 225 and 224, Appendix 1: PDF file including mean peak areas and associated SEM values for the features extracted from the raw data files.
