# Supplementary material for: Temperate Propolis Has Anti-Inflammatory Effects and Is a Potent Inhibitor of Nitric Oxide Formation in Macrophages
Source: Metabolites. 2020 Oct 14;10(10):413. doi: 10.3390/metabo10100413 (PMC7602400; doi:10.3390/metabo10100413)
Supplement: Supplementary file 1 [file metabolites-10-00413-s001.zip › metabolites-946794 supplementary/metabolites-946794 supplementary.docx]

Type of the Paper (Article)

**Temperate Propolis has Anti-inflammatory Effects and is a Potent Inhibitor of Nitric Oxide Formation in Macrophages**

S. Alanazi^1^, N. Alenzi^2^, J. Fearnley^3^, W. Harnett^4^* and D.G. Watson^4^*

^1.^ King Saud University, College of Applied Medical Sciences, Clinical Laboratory Sciences Department, Riyad, Saudi Arabia. [saalanazi@KSU.EDU.SA](mailto:saalanazi@KSU.EDU.SA) (SA)

^2^ Research and Laboratories Sector, National Drug and Cosmetic Control Laboratories (NDCCL), Saudi Food and Drug Authority, Riyad, Saudi Arabia. [NDenzi@sfda.gov.sa](mailto:NDenzi@sfda.gov.sa) (NA).

^3^ Apiceutical Research Centre, 6 Hunter Street, Whitby, North Yorkshire YO21 3DA, UK; james.fearnley@beearc.com (J.F.)

^4^Strathclyde Institute of Pharmacy and Biomedical Sciences, University of Strathclyde, 161, Cathedral Street, Glasgow G4 0RE., [w.harnett@strath.ac.uk](mailto:w.harnett@strath.ac.uk) (W.H.), [d.g.watson@strath.ac.uk](mailto:d.g.watson@strath.ac.uk) (D.G.W.)

***** Correspondence: d. g.watson@strath.ac.uk; Tel.: (+44141-548-2651) (D.G.W.); [w.harnett@strath.ac.uk](mailto:w.harnett@strath.ac.uk) +44141- 548-3725 (W.H.)

*Method of Analysis*

The propolis extracts to be used in testing the immune response were diluted to 100µg per mL and 20 µl was injected into the LS-MS LC-MS was carried out by using an Dionex 3000 pump connected to an Orbitrap Exactive mass spectrometer operated in positive/negative switching mode. The sheath gas and auxiliary gas were set at 50and 17 arbitrary units, respectively. The needle voltage was 4.5 kV in positive mode and 4.0 kV in negative mode. The heated capillary temperature was 275 °C. The HPLC was fitted with an ACE C18 column 150 × 4.6 mm, 3 μMparticle size (Hichrom, Reading, UK). Solvent A was 0.1% formic acid in water and solvent B was 0.1% formic acid in acetonitrile. The flow rate was 0.3 ml/min and the solvent gradient was as follows: 0 min 30% B, 30 min 100% B, 40 min 100% B, 41 min 30% B, 50 min 30% B. The flavonoids were identified according to their elemental composition with a mass deviation < 3 ppm from the calculated composition.

**Figure S1** Extracted ion traces for chrysin in samples 225 and 224.





**Figure S2** Extracted ion traces for pinocembrin in samples 225 and 224.





**Figure S3** Extracted ion traces for galangin in samples 225 and 224.





**Figure S4** Extracted ion traces for pinobanksin in samples 225 and 224.





**Figure S5** Extracted ion traces for galangin methyl ethers in samples 225 and 224.





**Figure S6** Extracted ion traces for kaempferol in samples 225 and 224.





| 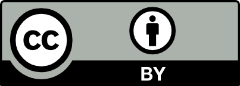 | © 2020 by the authors. Submitted for possible open access publication under the terms and conditions of the Creative Commons Attribution (CC BY) license (http://creativecommons.org/licenses/by/4.0/). |
| --- | --- |
