# Supplementary material for: Temperate Propolis Has Anti-Inflammatory Effects and Is a Potent Inhibitor of Nitric Oxide Formation in Macrophages
Source: Metabolites. 2020 Oct 14;10(10):413. doi: 10.3390/metabo10100413 (PMC7602400; doi:10.3390/metabo10100413)
Supplement: Supplementary file 1 [file metabolites-10-00413-s001.zip › metabolites-946794 supplementary/metabolites-946794-Appendix 1.pdf]

Data with SEM values and mean areas

m/z	Rt	Name	P LM	L/M	P224L	224/L	P225L	225/L	Mmean area	MSEM	Lmean area	LSEM	224 mean area	224SEM	225 mean area	225SEM
76.040	15.9	Glycine	0.005	2.367	0.000	7.222	0.000	8.452	44896	5766	106264	13680	767428	78815	898192	84780
90.055	14.8	L-Alanine	0.000	7.373	0.203	0.864	0.629	0.950	5658783	395461	41721815	3339620	36050554	2430632	39630466	2525586
90.055	15.4	L-Alanine	0.000	8.723	0.024	0.752	0.142	0.826	310321	14848	2706777	193837	2034733	161254	2236822	221188
104.071	18.2	4-Aminobutanoate	0.011	3.776	0.326	0.708	0.688	0.893	834512	226888	3151245	611565	2232624	646436	2814611	537968
104.107	14.5	Choline	0.000	4.826	0.000	0.407	0.000	0.379	33120157	1451347	159846224	12939802	65112172	5578133	60648475	5337327
104.107	4.7	Choline	0.000	0.307	0.077	2.198	0.021	2.899	1565702	30284	480426	63440	1056128	258734	1392652	278337
106.050	10.5	L-Serine	0.009	2.032	0.002	2.090	0.000	2.894	41242	8784	83813	9653	175151	16826	242555	12188
106.050	17.3	L-Serine	0.010	11.732	0.659	0.862	0.645	0.853	13028	3305	152850	35155	131793	29969	130304	31892
110.027	15.1	Hypotaurine	0.000	6.818	0.256	0.878	0.977	0.996	145768	6188	993811	74411	872208	67944	990175	96493
112.051	15.2	Cytosine	0.053	1.652	0.412	0.832	0.808	0.951	343108	63641	566904	78968	471697	78282	538957	79085
114.055	15.9	(S)-1-Pyrroline-5-carboxylate	0.000	2.539	0.003	0.558	0.014	0.680	1018052	153266	2584605	228907	1442071	96577	1756708	136666
114.066	14.8	Creatinine	0.000	5.664	0.590	0.944	0.364	1.109	919173	43925	5206234	409167	4916635	318375	5774334	434154
115.050	14.8	5,6-Dihydrouracil	0.000	8.423	0.227	0.859	0.489	0.924	187573	12351	1579961	141262	1356542	98249	1459594	87400
115.050	15.4	5,6-Dihydrouracil	0.000	12.434	0.002	0.523	0.013	0.665	9555	1352	118809	10303	62143	3951	79045	8052
116.071	16.0	L-Proline	0.000	12.175	0.000	0.172	0.001	0.359	281232	41845	3423940	301095	587943	67532	1230746	56906
118.086	11.3	L-Valine	0.012	1.651	0.021	1.439	0.013	1.500	15009043	1203683	24782685	2625392	35661567	2959067	37185661	3135859
120.066	14.5	L-Threonine	0.033	0.718	0.015	1.359	0.000	1.704	2015338	181587	1447225	136969	1967247	110073	2465523	102353
121.051	23.2	Purine	0.042	1.562	0.336	1.864	0.041	0.563	17899	2929	27950	3175	52111	22583	15729	4092
122.027	14.1	L-Cysteine	0.027	20.174	0.001	14.423	0.000	26.437	455	288	9175	2814	132336	16862	242559	28609
122.027	16.1	L-Cysteine	0.052	0.356	0.020	0.079	0.021	0.102	18620	4696	6631	1821	526	337	677	476
123.055	14.2	Nicotinamide	0.013	2.543	0.610	0.894	0.756	1.054	989799	39793	2517510	404150	2251689	301037	2654115	114162
123.055	24.3	Nicotinamide	0.050	2.939	0.234	2.923	0.024	2.167	88394	20758	259832	66685	759602	366430	562961	89848
126.022	14.8	Taurine	0.001	3.017	0.214	0.845	0.346	0.885	6184935	251863	18661782	1655443	15768562	1414277	16518938	1392534
127.050	12.7	Thymine	0.042	5.406	0.002	2.580	0.001	2.843	38377	9524	207466	62329	535322	37467	589817	62308
129.066	14.9	5,6-Dihydrothymine	0.000	2.407	0.187	0.879	0.470	1.088	1605777	183684	3865090	279427	3399049	161275	4207077	358238
131.034	14.6	Mesaconate	0.001	0.168	0.003	14.056	0.000	22.850	25558	3573	4303	2634	60478	10992	98311	10693
131.070	7.6	(S)-3-Methyl-2-oxopentanoic acid	0.040	0.602	0.443	1.289	0.227	1.452	903000	52357	543996	130584	701465	147230	789843	139534
132.077	14.8	Creatine	0.000	7.404	0.227	0.869	0.544	0.936	34337889	2147474	254235451	20950093	220927223	14863776	238074383	14807902
134.045	14.9	L-Aspartate	0.001	3.285	0.003	0.387	0.006	0.490	262513	34808	862229	102553	334056	30101	422282	38395
134.045	16.8	L-Aspartate	0.042	1.796	0.672	1.087	0.006	3.225	9793	2696	17584	1871	19118	2959	56711	8831
135.066	10.5	Dihydropteridine	0.021	0.333	0.002	6.949	0.002	8.877	15378	3148	5128	1206	35633	5127	45518	7125

136.062	5.2	Adenine	0.026	0.479	0.030	0.635	0.835	0.945	51875	8800	24848	2964	15782	1825	23472	5631
136.062	9.7	Adenine	0.000	4.250	0.000	1.708	0.000	2.160	406031	45874	1725616	143043	2946744	104038	3728148	242058
136.062	19.6	Adenine	0.005	0.450	0.321	1.192	0.064	1.480	89301	11101	40202	3784	47908	6246	59496	7960
137.046	8.3	Hypoxanthine	0.002	2.386	0.002	4.181	0.000	6.198	46427	10520	110756	11991	463122	64110	686472	60709
138.055	15.0	Anthranilate	0.011	0.553	0.001	27.497	0.002	24.970	40232	3929	22243	4200	611633	91216	555411	94187
142.026	16.0	Ethanolamine phosphate	0.003	2.367	0.454	0.895	0.811	1.038	460753	23248	1090508	118364	976413	84893	1132306	122304
146.092	15.3	4-Guanidinobutanoate	0.000	3.130	0.000	2.063	0.000	1.968	295593	64822	925259	85865	1908458	139846	1820812	132833
146.117	13.4	4-Trimethylammoniobutanoate	0.023	0.737	0.131	1.258	0.012	1.600	1780424	89072	1311322	142730	1650092	148026	2097512	203476
146.165	16.4	Spermidine	0.044	3.934	0.020	0.071	0.027	0.151	144786	69402	569654	157840	40314	5200	85968	44858
146.165	18.1	Spermidine	0.021	9.399	0.017	0.046	0.059	0.297	98247	11819	923425	249955	42485	5309	274473	161313
147.029	18.2	2-Oxoglutarate	0.013	#DIV/0!	0.015	0.035	0.014	0.020	0	0	32383	8607	1132	445	649	299
148.060	14.6	L-Glutamate	0.012	0.729	0.000	3.126	0.000	4.074	6482199	380682	4728757	420966	14779847	1102394	19265406	1522953
150.077	14.5	7-Methyladenine	0.036	3.436	0.000	11.588	0.004	13.448	9522	938	32714	8183	379075	38392	439934	82464
152.107	18.9	N-Methyltyramine	0.016	1.425	0.417	3.077	0.161	0.780	23476	2639	33459	2141	102958	78463	26111	4204
157.061	10.9	4-Imidazolone-5-propanoate	0.000	9.527	0.019	0.462	0.040	0.512	18286	3831	174215	7350	80515	28065	89145	30977
159.076	16.0	4-Methylene-L-glutamine	0.001	28.962	0.002	0.206	0.009	0.451	1218093	167358	35278398	4778695	7279878	627537	15896427	1190714
160.133	13.4	DL-2-Aminooctanoicacid	0.005	1.832	0.797	1.036	0.054	1.361	217498	15174	398489	41133	412671	34612	542168	50777
164.056	10.7	Pterin	0.009	24.471	0.544	1.195	0.003	2.156	1919	233	46966	10735	56142	9882	101236	7397
165.054	4.5	Phenylpyruvate	0.009	0.604	0.082	90.809	0.020	30.119	6988	701	4221	429	383327	174786	127139	36506
165.054	25.0	Phenylpyruvate	0.034	0.715	0.446	1.294	0.371	5.589	30829	2038	22043	2870	28532	7469	123202	102923
166.072	12.8	3-Methylguanine	0.000	66.900	0.001	2.155	0.000	3.805	38571	6156	2580387	269408	5561511	530733	9819280	981490
168.043	15.7	Taurocyamine	0.000	3.536	0.006	0.694	0.054	0.798	26327	3007	93082	5641	64580	6062	74324	6449
168.051	14.8	8-Hydroxyguanine	0.003	3.194	0.424	0.830	0.428	0.810	33037	4715	105505	14607	87544	15834	85461	19291
173.021	15.7	sn-Glycerol 3-phosphate	0.002	12.756	0.005	0.289	0.006	0.331	8970	2370	114420	17566	33103	3973	37847	7133
173.092	14.5	Glycylproline	0.044	0.499	0.000	4.187	0.000	4.711	189552	25013	94509	32431	395692	29620	445249	48989
173.092	11.3	Glycylproline	0.013	3.899	0.013	0.269	0.016	0.297	4292	645	16732	3337	4501	856	4975	711
176.103	16.0	L-Citrulline	0.001	31.765	0.002	0.199	0.009	0.432	449346	63803	14273662	2011394	2846276	259729	6159273	510846
180.086	18.1	D-Glucosamine	0.000	6.336	0.000	0.210	0.000	0.148	9472	1181	60013	6101	12589	2680	8872	1000
184.060	13.7	4-Pyridoxate	0.036	2.145	0.043	1.883	0.004	3.981	3155	433	6767	1282	12738	2137	26938	4146
184.060	9.9	4-Pyridoxate	0.007	3.487	0.000	12.459	0.000	26.905	2956	497	10310	1754	128451	11091	277381	32755
184.073	15.0	Choline phosphate	0.001	2.530	0.000	2.827	0.001	2.582	19093971	1007907	48308694	4550459	136592330	11212892	124725545	11552476
184.073	4.3	Choline phosphate	0.001	1.880	0.142	0.665	0.369	0.913	4725171	204228	8884193	658519	5910741	1656643	8108957	489092
185.081	20.3	3-Methoxy-4-hydroxyphenylethyleneglycol	0.030	1.479	0.036	0.655	0.126	0.726	34613	4458	51190	4819	33512	5468	37181	6779
185.128	18.3	N-(3-acetamidopropyl) pyrrolidin-2-one	0.014	3.260	0.014	0.292	0.032	0.429	15883	4609	51781	10095	15130	2639	22233	1501

186.112	9.7	Ecgonine	0.053	0.616	0.715	1.076	0.597	0.891	28743	3978	17708	2966	19060	2018	15780	1881
188.055	10.7	2-(Acetamidomethylene)succinate	0.016	#DIV/0!	0.001	9.431	0.001	12.793	0	0	2628	732	24784	3650	33618	4648
188.091	14.3	[FA hydroxy(4:0)] N-(3S-hydroxy-butanoyl)-homoserine lactone	0.053	2.075	0.073	6.045	0.018	8.052	11768	1058	24419	5019	147607	54428	196628	50091
188.103	14.0	5-guanidino-3-methyl-2-oxo-pentanoate	0.037	0.572	0.000	5.320	0.001	7.948	221510	31011	126739	23563	674288	56572	1007317	125454
189.087	14.6	N-Acetylglutamine	0.037	0.463	0.002	4.745	0.000	8.224	57685	10496	26681	6905	126609	18847	219427	24708
189.087	13.5	N-Acetylglutamine	0.015	0.221	0.000	14.599	0.000	21.852	100526	21643	22266	3096	325069	26573	486564	49315
189.160	22.2	N6,N6,N6-Trimethyl-L-lysine	0.016	1.776	0.990	0.998	0.134	1.268	1310533	118269	2327734	291525	2323237	180023	2951368	245653
190.089	4.7	Prenyl-L-cysteine	0.010	2.869	0.000	2.488	0.000	3.414	372805	85942	1069737	180546	2661100	240793	3652242	317903
190.118	22.4	L-Homocitrulline	0.000	#DIV/0!	0.000	0.014	0.000	0.031	0	0	507549	56963	6931	2506	15971	2986
191.066	18.2	Asp-Gly	0.009	#DIV/0!	0.009	0.013	0.009	0.006	0	0	34634	8373	437	282	206	206
191.114	22.5	N-(omega)-Hydroxyarginine	0.001	18030.784	0.002	0.056	0.002	0.157	170	170	3066791	462685	170612	15143	481033	50586
195.065	4.5	Ferulate	0.031	0.000	0.001	#DIV/0!	0.000	#DIV/0!	1990	672	0	0	864276	123060	140846	10027
196.083	9.9	2-Amino-4-hydroxy-6-hydroxymethyl-7,8-dihydropteridine	0.000	4.887	0.006	1.763	0.069	1.146	101575	5467	496399	29005	875386	87096	569055	19774
196.083	12.8	2-Amino-4-hydroxy-6-hydroxymethyl-7,8-dihydropteridine	0.003	74.610	0.001	3.399	0.000	4.533	1198	386	89413	16929	303952	37096	405285	42116
196.979	15.0	tetranitromethane	0.002	#DIV/0!	0.001	3.078	0.000	2.688	0	0	57668	10100	177495	19416	155004	14913
197.044	13.1	3-(3,4-Dihydroxyphenyl)pyruvate	0.041	3.518	0.026	0.184	0.168	0.501	12061	2688	42433	11173	7788	2963	21250	8716
198.027	16.3	Phosphoguanidinoacetate	0.002	0.165	0.000	129.853	0.000	180.746	2488	272	410	410	53244	5413	74111	8363
198.052	15.7	2-Amino-5-phosphopentanoic acid	0.002	11.538	0.006	0.313	0.009	0.364	23758	4547	274120	43928	85934	14286	99864	16149
203.085	4.9	Pyrene	0.019	5.515	0.065	1.691	0.006	2.559	7014	2394	38683	9429	65425	8798	98998	13895
203.091	18.2	Diethyl 2-methyl-3-oxosuccinate	0.011	16.732	0.011	0.060	0.015	0.170	23631	4362	395388	93519	23635	4003	67363	40850
203.106	9.6	Tremetone	0.044	1.377	0.046	0.689	0.437	2.656	12352	1433	17014	1434	11716	1813	45192	33322
203.139	12.7	Leu-Ala	0.002	3.235	0.473	1.202	0.206	1.474	19420	3605	62820	7978	75500	14775	92594	19644
204.086	16.7	N2-Acetyl-L-aminoadipate	0.007	0.643	0.034	1.671	0.451	1.168	27899	2255	17937	1808	29981	4187	20944	3329
204.123	11.1	O-Acetylcarnitine	0.000	4.658	0.002	0.490	0.002	0.483	7457687	389792	34740410	3303631	17032660	1589381	16764317	1664448
205.035	16.0	Oxaloglutarate	0.001	2.345	0.274	0.858	0.984	1.003	114892	5161	269379	25193	231111	21324	270170	29803
205.082	15.2	Glu-Gly	0.000	4.640	0.000	3.873	0.019	2.299	17088	2574	79283	7479	307098	24642	182310	30486
205.119	11.1	Triethylenemelamine	0.000	15.754	0.001	0.363	0.001	0.175	4498	1355	70857	7964	25725	4026	12418	1814
205.122	4.8	4'-Hydroxy-3'-prenylacetophenone	0.027	0.615	0.440	1.245	0.698	1.102	57863	6250	35589	5931	44324	9024	39224	6884
206.102	11.8	N-Acetyl-D-fucosamine	0.020	3.623	0.606	1.437	0.444	1.667	10604	1750	38422	8320	55198	29601	64058	30057
207.112	9.3	Phenylethylmalonamide	0.013	0.248	0.002	6.065	0.002	8.219	49152	10064	12200	2609	73997	10778	100267	15308
208.060	4.1	4-(2-Aminophenyl)-2,4-dioxobutanoate	0.000	0.449	0.973	0.992	0.196	0.766	1117780	74847	501402	61822	497640	87843	384094	57787
208.108	4.9	NNK (carcinogen)	0.044	2.253	0.018	0.287	0.458	2.940	35210	4966	79325	16563	22745	3764	233209	191058
209.117	4.9	Benzyl (2R,3S)-2-methyl-3-hydroxybutanoate	0.038	0.381	0.054	2.297	0.002	2.727	508306	109609	193901	62890	445323	93961	528839	33514
209.153	4.6	4-Heptyloxyphenol	0.028	0.312	0.794	0.912	0.326	1.948	553022	125491	172457	39608	157321	40109	335990	147117

210.087	15.7	4-Acetamido-2-amino-6-nitrotoluene	0.002	4.975	0.003	0.318	0.003	0.351	19610	2718	97561	13160	30995	5285	34279	6538
212.043	15.1	Phosphocreatine	0.001	2.157	0.006	1.768	0.002	2.739	1258829	58591	2715413	204187	4801520	493383	7436482	802800
213.040	15.1	2-Hydroxy-6-ketononatrienedioate	0.045	3.264	0.046	3.251	0.004	5.844	2469	379	8059	2105	26197	6897	47097	8127
213.098	14.9	Gly-His	0.028	0.526	0.939	1.028	0.226	1.988	682575	89034	358937	89879	369063	93686	713542	248429
213.098	15.7	Gly-His	0.019	9.536	0.027	0.183	0.028	0.193	271225	67664	2586349	681376	472051	16722	498922	85059
213.098	18.1	Gly-His	0.026	6.947	0.045	0.272	0.030	0.175	215552	33084	1497475	411126	407471	60256	262126	40734
213.123	4.9	Pro-Pro	0.009	1.758	0.090	1.915	0.008	2.153	10681	1800	18774	1729	35956	8174	40425	5361
216.063	15.7	sn-glycero-3-Phosphoethanolamine	0.002	8.118	0.006	0.332	0.008	0.372	248967	19873	2021113	309525	671127	81293	752044	113076
217.082	15.8	N3-(4-methoxyfumaroyl)-L-2,3-diaminopropanoate	0.049	1.419	0.022	0.609	0.469	1.225	18198	1943	25818	2727	15728	2526	31622	7028
217.155	4.9	Val-Val	0.002	0.461	0.333	0.811	0.005	1.735	72618	7575	33452	3632	27127	5005	58040	5484
218.138	9.9	O-Propanoylcarnitine	0.000	0.321	0.000	2.871	0.000	2.967	1631658	83215	523810	70611	1503739	144235	1554160	151688
218.186	27.5	Deoxyhypusine	0.029	11.573	0.459	1.611	0.044	2.234	31451	6818	363992	109220	586484	261081	813301	156923
219.080	4.8	Cys-Pro	0.000	4.449	0.003	2.697	0.000	4.054	8604	2045	38278	3891	103230	13203	155197	13566
219.097	14.6	L-Ala-L-Glu	0.000	47.907	0.000	0.328	0.000	0.204	5623	1258	269364	19163	88364	13852	55036	5825
219.097	9.9	L-Ala-L-Glu	0.001	162.066	0.001	9.411	0.001	17.837	188	188	30505	4785	287067	36380	544097	65202
219.122	13.0	3-Hydroxysebacicacid	0.043	1.719	0.041	0.597	0.444	3.271	43354	8393	74518	10393	44516	6971	243746	203272
219.138	9.9	[FA (14:5)] 5,7,9,11,13-tetradecapentaenoic acid	0.006	0.052	0.001	20.746	0.009	15.503	436639	91331	22617	3077	469204	70466	350631	78360
220.081	13.7	O-Succinyl-L-homoserine	0.009	8.035	0.003	4.086	0.000	7.641	995	329	7995	1731	32668	4875	61091	5840
221.096	4.3	Met-Ala	0.000	0.096	0.382	2.692	0.219	2.435	1381041	52851	132827	34636	357567	232665	323440	133612
221.153	9.9	Oblongolide	0.001	1.603	0.367	2.601	0.032	0.716	18766	923	30074	1938	78232	48489	21522	2754
222.097	13.9	N-Acetyl-D-glucosamine	0.003	9.945	0.071	0.578	0.246	0.742	6092	1218	60589	10457	34994	6665	44933	7001
222.098	12.7	N-Acetyl-D-glucosamine	0.018	21.617	0.000	5.012	0.003	6.840	873	392	18878	5220	94619	10274	129131	21198
223.111	4.3	S-Butyl-DL-homocysteine-[S,R]-sulfoximine	0.001	0.193	0.615	1.503	0.840	1.209	1592759	50803	306745	191035	461027	227398	370791	241928
224.164	27.4	Tigloidine	0.005	0.682	0.388	3.150	0.207	0.838	49949	1890	34083	3606	107377	77450	28563	1676
224.164	21.8	Tigloidine	0.040	0.479	0.116	1.572	0.358	7.830	55377	10497	26510	2978	41667	7791	207588	178705
224.164	8.5	Tigloidine	0.050	2.038	0.183	0.615	0.042	0.464	23351	2871	47591	9431	29272	8632	22097	2954
225.148	21.1	[FA] Methyl jasmonate	0.038	1.447	0.618	0.913	0.390	4.901	23313	2250	33737	3594	30818	4373	165340	139825
227.113	15.7	Carnosine	0.003	4.115	0.006	0.378	0.008	0.422	143396	16446	590020	83364	223227	24296	248961	27413
228.098	9.7	Deoxycytidine	0.006	5.061	0.172	1.391	0.000	2.593	12845	8013	65009	12000	90451	12461	168556	9884
228.128	7.5	ametryn	0.017	0.289	0.073	2.030	0.032	6.734	10555	2186	3052	726	6196	1327	20554	5978
229.070	10.7	2-Hydroxy-3-carboxy-6-oxo-7-methylocta-2,4-dienoate	0.003	6.122	0.000	107.289	0.000	122.692	263	263	1613	86	173044	17297	197887	18483
229.101	4.3	Chrysene	0.009	0.211	0.095	1.775	0.650	1.222	29161	5865	6147	2275	10913	855	7512	1824
229.101	10.7	Chrysene	0.003	2.631	0.900	1.044	0.100	1.753	4251	1069	11183	1395	11670	3468	19607	4129
229.118	14.3	(S)-ATPA	0.017	0.282	0.101	2.846	0.014	4.943	689018	141215	194461	24932	553502	178568	961314	208129

229.179	4.5	[FA oxo(13:0)] 2-oxo-tridecanoic acid	0.050	0.313	0.975	0.993	0.801	1.161	48813	13059	15272	2562	15169	1892	17729	8946
230.095	14.9	Ergothioneine	0.001	2.602	0.683	1.061	0.140	1.260	168991	10845	439734	38205	466706	51180	554111	59028
230.247	14.6	[SP (14:0)] 1-deoxy-tetradecasphinganine	0.039	0.611	0.327	4.647	0.645	1.154	83344	8642	50963	10482	236838	171019	58812	12764
231.032	15.1	hydroxphenylpropionic acid sulfate	0.022	0.513	0.227	0.625	0.043	0.569	68863	10401	35302	5417	22066	8580	20099	3324
232.083	14.2	N-Succinyl-L-glutamate 5-semialdehyde	0.001	2.585	0.271	1.174	0.099	1.265	22372	2919	57832	5507	67920	6649	73164	6342
232.154	8.9	O-Butanoylcarnitine	0.026	1.662	0.045	0.663	0.134	0.740	242970	31504	403713	50280	267614	25948	298790	39593
234.077	15.6	2-Hydroxy-6-oxo-(2'-aminophenyl)-hexa-2,4-dienoate	0.030	6.614	0.059	0.310	0.153	0.520	870689	100868	5759028	1633487	1785233	369258	2995614	260938
234.077	18.1	2-Hydroxy-6-oxo-(2'-aminophenyl)-hexa-2,4-dienoate	0.001	9.505	0.004	0.386	0.005	0.407	558581	96714	5309372	700032	2052066	268146	2161004	453547
235.027	17.0	trihydroxy phenylethanol sulfate isomer	0.028	#DIV/0!	0.000	13.547	0.001	20.606	0	0	4493	1465	60863	6699	92577	12990
235.092	18.0	Glu-Ser	0.006	12.149	0.006	0.116	0.007	0.134	2916	501	35423	6990	4098	516	4762	752
235.205	24.4	[FA (16:3)] 4,6,11-hexadecatrienal	0.000	0.461	0.136	1.384	0.010	1.337	34446	2516	15872	1150	21963	3365	21229	1245
236.998	18.2	Fenpiclonil	0.000	96.673	0.000	0.189	0.000	0.122	26936	4843	2603994	166296	492373	46838	316416	32605
237.148	4.4	4-Heptyloxybenzoic acid	0.029	0.190	0.117	1.780	0.145	1.818	235369	62873	44714	6962	79609	17974	81306	20752
237.148	20.4	4-Heptyloxybenzoic acid	0.007	1.671	0.894	1.029	0.373	0.857	21665	2176	36201	3519	37244	6685	31017	4288
237.221	3.9	[FA (16:2)] 6,11-hexadecadienal	0.033	0.516	0.000	32.606	0.000	20.986	26986	4505	13936	1055	454383	28727	292449	18325
239.114	22.1	quinonoid dihydro-(6H)-biopterin	0.004	0.511	0.045	0.441	0.518	1.178	191287	9142	97774	19911	43082	11460	115200	16676
239.164	17.1	[FA oxo(5:1/5:0/4:0)] (1R,2R)-3-oxo-2-(2'Z-pentenyl)-cyclopentanebutanoic acid	0.003	1.522	0.319	1.241	0.369	4.565	19770	2126	30080	1020	37336	6518	137308	108530
240.109	12.8	Dihydrobiopterin	0.001	27.752	0.002	2.914	0.001	4.008	20376	2647	565488	69389	1647779	200055	2266251	245462
241.031	16.1	L-Cystine	0.009	0.492	0.005	0.108	0.005	0.159	420341	52174	206817	38726	22406	4447	32784	14700
241.129	14.3	Homocarnosine	0.003	2.860	0.152	1.377	0.000	2.594	15580	2446	44560	5712	61376	9022	115578	8411
241.154	12.9	Slaframine	0.003	0.475	0.226	1.681	0.209	1.691	140523	15552	66783	9700	112231	32221	112916	31316
241.154	24.5	Slaframine	0.051	0.635	0.334	6.951	0.670	1.151	44593	4467	28324	5742	196886	157488	32597	7815
241.179	15.9	[FA oxo(5:2/5:0/4:0)] (1S,2S)-3-oxo-2-pentyl-cyclopentanebutanoic acid	0.027	0.663	0.850	1.045	0.166	1.207	36113	3956	23932	1831	24999	5099	28892	2728
242.113	9.5	5-Methyl-2'-deoxycytidine	0.044	1.574	0.038	2.053	0.010	3.633	3832	427	6034	806	12386	2278	21922	4039
242.175	17.2	Valeroidine	0.018	0.651	0.111	1.396	0.095	1.346	20791	1833	13543	1785	18912	2465	18235	1807
243.101	4.0	Equol	0.044	0.057	0.000	55.063	0.004	54.359	2957	1047	168	168	9265	1140	9147	1833
243.159	26.7	2-isocapryloyl-3R-hydroxymethyl-γ-butyrolactone	0.011	1.488	0.030	0.659	0.332	0.853	17906	778	26643	2320	17545	2740	22718	3050
243.159	8.5	2-isocapryloyl-3R-hydroxymethyl-γ-butyrolactone	0.033	0.651	0.552	1.146	0.385	2.672	21328	2201	13881	2048	15909	2577	37096	24331
245.059	14.2	penem CGP31608	0.000	4.508	0.001	7.592	0.000	9.956	41053	3458	185085	11345	1405138	156830	1842771	200141
245.059	12.9	penem CGP31608	0.003	6.589	0.000	26.567	0.000	36.561	2145	735	14134	2322	375494	36177	516760	52969
245.149	10.9	N-hexenoylglutamine	0.008	2.240	0.661	1.151	0.448	1.296	16709	2795	37424	5176	43076	11219	48497	12703
246.170	8.1	N-(octanoyl)-L-homoserine	0.004	1.425	0.002	0.672	0.001	0.630	241734	17310	344487	21279	231620	15105	217136	12154
247.054	14.2	dihydrocamalexate	0.011	#DIV/0!	0.001	24.495	0.001	38.842	0	0	1502	379	36800	5013	58353	8437

247.111	4.3	Met-Pro	0.009	0.252	0.163	1.593	0.542	0.768	105638	19858	26625	8944	42409	4994	20454	3437
247.129	12.4	Glu-Val	0.002	3.967	0.913	0.981	0.327	0.798	23388	3063	92787	11770	91052	9964	74048	13814
247.140	14.3	N2-(D-1-Carboxyethyl)-L-arginine	0.002	4.929	0.000	6.081	0.000	10.833	106169	22957	523354	72330	3182756	308356	5669642	537848
248.149	11.5	Hydroxybutyrylcarnitine	0.000	7.752	0.001	0.346	0.001	0.367	30241	3209	234410	21837	81018	7000	85957	8733
249.127	4.6	Met-Val	0.009	0.508	0.151	1.523	0.101	1.698	39636	3239	20133	4849	30653	4722	34188	6033
249.221	4.1	[FA (17:3)] 8Z,11Z,14Z-heptadecatrienal	0.004	0.450	0.000	36.226	0.001	71.177	12600	1453	5669	517	205370	17413	403510	55011
250.093	16.5	S-Acetyldihydrolipoamide	0.002	13.455	0.001	2.313	0.001	3.498	91354	5882	1229164	181828	2842820	294649	4300168	467411
250.093	7.9	S-Acetyldihydrolipoamide	0.027	7.498	0.005	3.275	0.008	2.908	2679	902	20083	5667	65781	10489	58405	9392
251.054	17.2	2-Hydroxy-6-oxo-6-(2-hydroxyphenoxy)-hexa-2,4-dienoate	0.000	3.642	0.079	1.937	0.088	1.824	4622	715	16834	1499	32606	7155	30711	6538
251.069	14.1	gamma-L-Glutamyl-L-cysteine	0.001	14.204	0.000	8.063	0.001	14.927	7116	1201	101077	12701	815024	89310	1508823	180846
251.106	4.0	Met-Thr	0.034	0.455	0.003	18.217	0.003	58.776	8839	1630	4020	974	73223	12471	236256	42487
251.143	4.3	(R)-4'-Deoxyindenestrol	0.000	0.115	0.004	7.071	0.034	3.750	1475621	57312	170071	37342	1202620	213238	637737	162078
251.164	20.8	Xanthoxin	0.020	1.993	0.113	6.517	0.271	3.352	22307	3344	44447	6663	289653	127468	148971	84429
252.109	8.3	Muramic acid	0.004	2.651	0.043	0.636	0.464	1.127	62242	7352	165003	21840	104874	11562	185978	16644
253.067	18.1	Met-Cys	0.025	#DIV/0!	0.026	0.017	0.025	0.000	0	0	28470	8993	484	306	0	0
253.122	4.0	cis-Hinokiresinol	0.007	0.540	0.001	2.434	0.038	1.765	106483	11354	57481	8939	139882	13225	101438	15252
253.144	9.7	ubiquinol-1	0.036	9.683	0.038	0.119	0.033	0.083	26606	3494	257634	80985	30677	5540	21364	4435
253.216	4.0	[FA (16:2)] 9,12-hexadecadienoic acid	0.032	0.540	0.686	0.915	0.760	1.090	158338	24631	85511	12984	78232	11705	93167	20464
255.097	24.3	N-Ribosylnicotinamide	0.008	8.968	0.013	4.839	0.136	2.668	2217	254	19880	4199	96189	20505	53035	18520
255.231	16.8	(9Z)-Hexadecenoic acid	0.041	0.516	0.558	1.154	0.840	0.927	55912	9414	28824	6175	33265	3855	26711	8085
256.030	18.2	Pronamide	0.000	#DIV/0!	0.000	0.034	0.000	0.012	0	0	108502	8698	3743	856	1317	478
258.110	14.5	sn-glycero-3-Phosphocholine	0.000	5.060	0.001	0.427	0.001	0.400	154140356	8190520	779997098	71953535	332676294	34133894	311927335	32216331
259.092	12.7	(1-Ribosylimidazole)-4-acetate	0.004	2.981	0.000	4.365	0.001	3.943	19026	2832	56714	8137	247567	19018	223618	24043
260.113	14.5	Proacacipetalin	0.000	5.233	0.001	0.412	0.001	0.385	1588049	88040	8310740	761434	3420262	343255	3201530	328765
260.197	20.5	Leu-Lys	0.000	0.500	0.221	1.198	0.353	1.155	517854	17899	258772	29806	309963	25434	298898	28370
261.148	10.8	Lacinilene C 7-methyl ether	0.023	3.709	0.490	0.793	0.142	0.532	120506	69498	446989	97204	354273	85201	237681	88342
262.103	15.8	Ala-Asp-Gly	0.008	3.427	0.006	1.992	0.020	1.683	35266	7246	120869	20802	240826	26515	203393	21421
265.111	21.0	2-(6'-methylthio)hexylmalate	0.040	1.707	0.048	1.426	0.170	1.299	993775	102785	1696732	255159	2419001	187043	2203439	228529
265.252	3.8	[FA (18:2)] 9,12-octadecadienal	0.016	6.187	0.000	3.001	0.677	1.172	17369	2791	107455	25447	322514	31573	125884	34462
266.095	14.5	Albendazole	0.001	147.364	0.001	0.097	0.002	0.072	2952	1955	435001	66402	42309	19378	31235	8397
266.138	8.4	N(alpha)-Benzyloxycarbonyl-L-leucine	0.009	2.273	0.484	1.146	0.287	1.268	18470	1959	41985	5869	48107	6052	53257	8051
267.119	18.2	5'-amino-5'-deoxyadenosine	0.024	10.102	0.055	0.297	0.035	0.193	7151	1197	72245	20362	21436	4981	13910	2684
267.195	4.2	[PR] Juvenile hormone III	0.022	0.104	0.166	5.781	0.058	10.988	609474	166518	63126	12055	364952	186207	693613	257972
269.044	18.1	Coumestrol	0.001	#DIV/0!	0.001	0.015	0.001	0.004	0	0	68536	9884	1050	347	299	299

269.080	4.0	Homolanthionine	0.033	0.253	0.002	1403.812	0.002	671.782	2951	758	746	341	1047492	166929	501268	82381
269.099	18.2	8-Azaadenosine	0.007	12.508	0.012	0.205	0.011	0.166	3446	485	43098	9118	8820	2036	7158	1118
269.174	10.1	dihydroartemisinic acid hydroperoxide	0.042	0.703	0.062	1.696	0.315	2.713	54350	5759	38208	3477	64798	11049	103641	58487
272.078	7.6	hydroxypyridine glucuronide	0.035	#DIV/0!	0.001	9.241	0.006	8.401	0	0	4354	1521	40231	5506	36574	7307
272.221	4.4	Tridecanoylglycine	0.051	0.138	0.116	1.828	0.311	8.215	224624	75805	31079	7756	56802	12468	255321	198884
273.036	16.3	α-(2,6-anhydro-3-deoxy-D-arabino-heptulopyranosid)onate 7-phosphate	0.003	#DIV/0!	0.745	0.909	0.014	1.981	0	0	31497	6011	28633	6119	62404	8211
273.091	5.1	Thienamycin	0.013	2.912	0.232	1.389	0.204	1.479	4726	747	13762	2455	19110	3376	20358	4100
275.134	15.4	ala-gly-ala-gly	0.017	3.211	0.000	38.341	0.000	88.965	1237	277	3971	794	152268	15712	353320	30305
276.155	17.1	L-a-glutamyl-L-Lysine	0.013	0.668	0.505	0.878	0.353	1.205	195684	8588	130673	18005	114668	14436	157463	20743
276.180	9.2	[FA hydroxy(10:0)] N-(3S-hydroxydecanoyl)-L-serine	0.004	11.884	0.004	0.091	0.005	0.124	4215	576	50089	9046	4537	725	6195	912
278.024	18.1	Fenitrothion	0.014	#DIV/0!	0.019	0.076	0.016	0.036	0	0	59654	16170	4541	1209	2139	723
278.105	14.2	Metazachlor	0.037	#DIV/0!	0.020	7.331	0.008	11.738	0	0	5060	1789	37093	9572	59391	12778
279.137	4.1	Pantetheine	0.025	0.310	0.258	1.846	0.340	1.601	54111	11976	16800	5685	31015	10165	26893	8245
281.247	4.0	Linoleate	0.019	0.208	0.093	3.153	0.223	1.398	656686	152214	136691	20149	430949	141975	191054	35832
282.095	14.5	[GL] 1-acyl-3-O-beta-D-galactosyl-sn-glycerol	0.002	2.039	0.104	0.800	0.042	0.734	604167	44642	1231654	112799	984871	76169	903854	80816
283.114	18.2	2-Aminoadenosine	0.003	13.934	0.005	0.169	0.004	0.150	6036	1203	84108	14703	14187	1642	12615	2706
283.190	15.9	12-trans-Hydroxy juvenile hormone III	0.015	0.577	0.130	1.697	0.306	4.466	28881	3414	16676	1948	28302	6324	74470	50669
283.190	29.1	12-trans-Hydroxy juvenile hormone III	0.039	0.634	0.425	1.233	0.377	1.220	27243	3513	17277	2016	21304	4307	21083	3535
284.098	12.7	Guanosine	0.046	13.235	0.001	23.082	0.000	29.869	691	312	9141	3194	210981	31787	273025	31843
286.274	7.5	[SP (17:0)] heptadecasphing-4-enine	0.022	0.305	0.408	0.720	0.469	1.514	37901	8177	11566	3103	8323	2075	17515	7117
286.310	9.1	[SP] 1-deoxy-sphinganine	0.000	0.050	0.038	6.990	0.004	12.958	351267	33913	17697	3921	123710	38037	229319	43101
290.145	18.3	Asp-Arg	0.001	9.928	0.046	0.612	0.475	0.867	11858	2093	117724	16947	72074	8896	102018	12534
291.129	16.8	N-(L-Arginino)succinate	0.002	24.687	0.002	3.091	0.001	5.223	1996	308	49283	8052	152315	19077	257384	32061
292.102	13.2	2,7-Anhydro-alpha-N-acetylneuraminic acid	0.011	2.197	0.002	0.192	0.002	0.279	223064	23733	490149	70312	93925	7804	136811	35036
292.102	17.5	2,7-Anhydro-alpha-N-acetylneuraminic acid	0.000	0.218	0.031	2.313	0.034	2.375	1442950	148203	314527	118731	727578	114139	746926	129380
292.113	15.8	Ala-Asp-Ser	0.018	26.499	0.012	2.608	0.001	4.340	305	305	8075	2258	21060	3405	35045	4789
296.065	14.6	Aminoimidazole ribotide	0.000	2.787	0.111	0.817	0.042	0.763	5580422	355619	15550577	1277033	12711386	984676	11858300	887359
296.116	15.5	Triadimenol	0.000	30.775	0.001	2.416	0.001	3.533	5575	1183	171573	18839	414566	38457	606136	62114
296.116	16.1	Triadimenol	0.028	#DIV/0!	0.033	3.601	0.004	6.598	0	0	15760	5154	56748	14111	103982	18044
296.258	3.9	[SP (3:0)] sphinga-4E,8E,10E-trienine	0.033	0.174	0.018	10.425	0.001	5.368	25848	7337	4503	1618	46940	12381	24170	3513
298.096	7.6	5'-Methylthioadenosine	0.012	2.125	0.004	1.713	0.000	2.085	1516901	251031	3223020	454861	5519649	408463	6720519	495846
298.096	16.5	5'-Methylthioadenosine	0.001	16.085	0.002	2.271	0.001	3.484	32774	4772	527168	77023	1197229	129681	1836495	218724
299.221	4.1	[FA (17:1/2:0)] 8E-Heptadecenedioic acid	0.003	0.402	0.359	9.691	0.576	1.195	80646	9623	32396	7619	313949	278891	38700	7803

300.120	11.1	[PC acety] 1-acetyl-sn-glycero-3-phosphocholine	0.001	#DIV/0!	0.001	0.270	0.004	8.912	0	0	3698	474	998	317	32962	5845
300.289	7.5	[SP] 3-dehydrosphinganine	0.037	0.729	0.767	1.048	0.673	1.067	858867	73352	626294	62060	656069	75339	668119	73410
301.152	21.0	Ala-Asn-Pro	0.042	3.245	0.122	0.509	0.179	0.573	13970	2614	45332	11634	23083	4705	25976	5852
308.064	15.3	dCMP	0.000	23.917	0.000	0.091	0.000	0.076	3010	818	72000	7817	6558	1381	5446	651
308.090	14.2	Glutathione	0.000	3.903	0.001	6.268	0.000	8.205	3975018	164749	15514619	1304003	97247820	11072519	127301628	14142938
309.242	4.0	[FA methyl(18:2)] methyl 9,10-epoxy-12,15-octadecadienoate	0.008	9.137	0.014	0.238	0.049	0.447	12957	1452	118387	24957	28146	6397	52893	11489
310.113	13.2	N-Acetylneuraminate	0.013	2.197	0.002	0.170	0.004	0.297	215484	22675	473444	70792	80249	7981	140781	30232
311.164	9.3	[Fv] Dihydrocordoin	0.053	0.614	0.474	1.187	0.351	6.241	83874	11934	51535	8171	61191	10050	321645	262844
311.164	19.3	[Fv] Dihydrocordoin	0.018	1.566	0.687	1.370	0.499	2.147	26106	4354	40886	1935	56004	35320	87786	64347
311.221	4.1	[FA (18:3)] 13S-hydroperoxy-9Z,11E,14Z-octadecatrienoic acid	0.049	0.468	0.000	86.842	0.000	98.895	36963	7564	17316	3182	1503790	92168	1712511	128110
311.221	26.3	[FA (18:3)] 13S-hydroperoxy-9Z,11E,14Z-octadecatrienoic acid	0.046	0.632	0.410	2.395	0.854	1.039	31620	4299	19987	2333	47869	30927	20765	3392
311.257	3.8	Methoprene	0.000	4.448	0.000	0.497	0.000	0.276	304609	117640	1354855	66659	673074	100006	373371	95801
313.029	16.3	gamma-Glutamyl-Se-methylselenocysteine	0.007	#DIV/0!	0.054	0.438	0.118	0.563	0	0	35338	7923	15479	2691	19898	3424
313.273	3.9	[FA oxo(19:0)] 10-oxo-nonadecanoic acid	0.002	2.198	0.065	0.878	0.013	0.866	863529	197668	1898179	69728	1667167	85968	1642893	34672
313.273	18.2	[FA oxo(19:0)] 10-oxo-nonadecanoic acid	0.045	1.713	0.543	1.850	0.012	0.605	15583	3880	26689	2768	49386	34700	16142	1931
315.079	18.0	Valdecoxib	0.010	111.227	0.010	0.021	0.011	0.040	586	371	65233	15972	1368	458	2584	600
324.058	15.2	CMP	0.003	2.389	0.791	1.041	0.072	1.341	138707	6508	331320	35659	345013	35358	444387	43110
324.059	16.3	CMP	0.000	8.661	0.021	1.532	0.005	2.146	17974	2177	155686	17187	238479	24241	334145	39753
325.162	18.2	Procollagen 5-(D-galactosyloxy)-L-lysine	0.005	10.943	0.010	0.254	0.006	0.122	4515	550	49409	9474	12528	2933	6030	637
325.200	14.6	[FA oxo,hydroxy(18:3)] 12-oxo-14,18-dihydroxy-9Z,13E,15Z-octadecatrienoic acid	0.013	0.437	0.970	1.006	0.434	1.185	49718	7725	21744	2796	21881	2165	25763	4023
325.273	3.8	[FA hydroxy(20:2)] 11R-hydroxy-12E,14Z-eicosadienoic acid	0.000	8.678	0.000	0.427	0.000	0.361	68941	15840	598243	39280	255516	50510	215713	22412
327.252	11.6	[FA methoxy,hydroxy(18:2)] 8-methoxy-13-hydroxy-9,11-octadecadienoic acid	0.022	0.545	0.212	3.117	0.560	1.177	36766	5122	20046	2711	62483	29617	23597	5159
327.288	3.8	2-Oxophytanate	0.009	3.140	0.151	0.699	0.331	0.734	73242	23740	229948	39715	160798	14556	168742	44794
331.249	3.9	[FA trihydroxy(18:0)] 9S,12S,13S-trihydroxy-10E-octadecenoic acid	0.024	9.046	0.015	2.152	0.022	2.523	5873	2326	53132	14907	114359	14501	134053	24477
332.146	14.3	Thr-Asp-Pro	0.002	3.657	0.332	0.851	0.527	1.105	21168	1783	77411	9698	65905	5458	85568	7789
333.188	18.9	Ala-Ser-Arg	0.042	2.033	0.152	0.669	0.208	0.673	28564	3227	58083	10905	38855	4820	39094	8870
335.073	16.0	sn-glycero-3-Phospho-1-inositol	0.001	22.162	0.004	0.432	0.179	0.776	3624	804	80318	9725	34692	4759	62322	7719
335.257	3.8	5-alpha-THDOC	0.010	12.648	0.383	0.752	0.066	1.570	9781	1924	123715	28386	93072	17400	194212	17211
336.087	14.6	S-Formylglutathione	0.000	4.273	0.000	0.536	0.000	0.518	224215	9554	958163	43590	513111	36616	496044	41193
336.139	15.9	N4-(Acetyl-beta-D-glucosaminyl)asparagine	0.006	3.659	0.002	0.031	0.002	0.105	9489	1329	34714	5635	1091	564	3630	1147
337.273	3.8	5beta-Pregnane-3alpha,17alpha,20alpha-triol	0.000	4.826	0.000	0.439	0.000	0.430	623067	30295	3007178	230207	1320415	95387	1291997	76098
337.273	20.1	5beta-Pregnane-3alpha,17alpha,20alpha-triol	0.021	0.600	0.202	3.211	0.001	1.561	20917	2563	12551	1182	40298	18860	19598	775

338.076	15.9	5-Hydroxymethyldeoxycytidylate	0.002	7.965	0.004	0.276	0.003	0.257	23791	4448	189488	28481	52245	8030	48781	9399
338.174	18.4	Olopatadine	0.016	16.782	0.033	0.229	0.034	0.235	5147	1101	86373	22932	19788	5116	20308	3505
340.197	14.1	Leu-Ala-His	0.000	0.171	0.081	0.539	0.026	0.372	40403	3931	6897	1405	3715	703	2568	520
341.304	3.8	[FA oxo(21:0)] 2-oxo-heneicosanoic acid	0.000	3.496	0.000	0.540	0.000	0.412	627204	19145	2192753	118322	1184112	82770	904062	80419
341.304	7.5	[FA oxo(21:0)] 2-oxo-heneicosanoic acid	0.001	2.202	0.018	0.704	0.837	0.948	43593	5274	95983	8159	67609	5316	91008	21704
344.279	4.9	1,2-dioctanoyl-1-amino-2,3-propanediol	0.000	4.596	0.001	2.197	0.001	2.255	28344	2356	130268	6714	286237	25776	293781	26458
347.167	23.5	Asp-Gly-Arg	0.024	1.441	0.373	4.076	0.407	3.082	21289	2692	30675	2230	125045	96390	94541	70533
348.070	13.7	AMP	0.007	3.809	0.008	1.801	0.003	1.945	242566	25077	923972	156164	1664380	158121	1797161	159602
348.070	16.6	AMP	0.035	4.217	0.007	3.455	0.007	5.228	2332	272	9833	2623	33973	5787	51404	9719
349.117	12.9	Camptothecin	0.001	2.661	0.000	9.794	0.000	13.505	1503306	41165	4000209	397792	39176354	3375732	54021306	5395945
350.120	12.9	Thr-Asp-Asp	0.001	2.879	0.000	10.976	0.000	15.285	149506	5497	430502	41529	4725366	418718	6580409	657443
351.113	12.9	Biapenem	0.000	4.167	0.000	11.420	0.000	16.182	31159	3719	129849	10280	1482900	133610	2101164	209509
352.116	12.9	Met-Asp-Ser	0.000	8.396	0.000	66.740	0.000	91.330	299	298	2507	169	167295	16185	228933	23207
353.304	3.8	auricolate	0.000	6.302	0.000	0.375	0.000	0.410	150549	20209	948792	54846	356156	32947	389118	14861
354.336	4.2	[FA (20:0)] N-(11Z-eicosaenoyl)-ethanolamine	0.045	0.133	0.085	3.038	0.322	3.159	1225632	400600	163343	90053	496170	144560	516073	312862
357.249	21.9	Leu-Lys-Pro	0.021	2.480	0.402	1.210	0.621	1.161	24380	5088	60469	11120	73169	9291	70227	15451
361.273	3.8	[ST (3:0)] (5Z,7E)-(1S,3R)-24-nor-9,10-seco-5,7,10(19)-cholatriene-1,3,23-triol	0.000	0.301	0.000	0.398	0.001	0.439	9610259	478892	2891784	249356	1149724	105305	1270854	83361
363.288	3.7	[ST (3:0)] 24-Nor-5beta-chol-22-ene-3alpha,7alpha,12alpha-triol	0.001	2.697	0.000	0.309	0.000	0.352	1501676	59505	4050195	342796	1252031	108677	1425781	98733
363.325	4.0	[ST (2:0)] 5beta-Cholane-3alpha,24-diol	0.048	0.526	0.405	1.309	0.656	1.195	9046	1137	4755	1499	6226	721	5680	1342
365.304	3.7	[GL (8:0)] 2-(8-[3]-ladderane-octanyl)-sn-glycerol	0.000	4.956	0.000	0.260	0.000	0.267	681098	21299	3375280	260737	878046	75691	899932	56081
365.340	4.0	[FA (24:2)] 5,9-tetracosadienoic acid	0.020	0.225	0.002	12.382	0.003	6.876	6643	1044	1497	1496	18533	3354	10292	1695
367.209	18.2	6alpha,9-Difluoro-11beta-hydroxypregn-4-ene-3,20-dione	0.028	6.348	0.039	0.237	0.029	0.167	6249	1364	39672	10949	9404	2348	6616	975
369.335	3.9	[FA oxo(23:0)] 2-oxo-tricosanoic acid	0.003	5.404	0.032	0.526	0.008	0.363	11339	2628	61278	9781	32232	5295	22261	4209
377.304	3.8	3alpha-Hydroxy-5beta-cholanate	0.000	2.654	0.000	0.196	0.000	0.222	363066	13494	963511	76879	189066	18157	214231	9992
379.320	3.8	[ST (3:0/3:0)] (7E)-(1R,3R)-9,11,21-trinor-9,10-seco-5,7-cholestadien-1,3,25-triol	0.000	5.875	0.000	0.142	0.000	0.192	53384	15127	313638	19361	44434	10828	60357	15430
380.111	12.1	(R)-S-Lactoylglutathione	0.001	#DIV/0!	0.000	7.408	0.000	10.455	0	0	14631	2203	108382	7974	152968	12671
381.224	7.5	4,4-Difluoro-17beta-hydroxyandrost-5-en-3-one propionate	0.014	0.591	0.518	1.227	0.766	0.880	31014	3021	18323	3016	22484	5362	16126	6427
384.114	14.4	Succinyladenosine	0.050	#DIV/0!	0.006	3.782	0.006	2.794	0	0	12408	4814	46931	8046	34669	4234
385.128	13.7	4'-Demethyldeoxypodophyllotoxin	0.015	2.026	0.000	3.021	0.000	3.160	22747	3570	46082	6596	139230	10116	145611	9448
385.219	13.1	Lys-Thr-His	0.009	12.948	0.011	2.345	0.003	2.999	2147	347	27794	6264	65186	9674	83346	11276
386.289	4.9	3-Hydroxy-cis-5-tetradecenoylcarnitine	0.002	9.282	0.107	1.459	0.000	6.815	3070	800	28494	4529	41569	5769	194180	20226
387.288	3.8	Testosterone isocaproate	0.004	1.806	0.000	0.134	0.000	0.163	10422315	501428	18821217	1710605	2525513	215597	3060405	207952
388.292	3.8	Leu-Lys-Lys	0.004	1.811	0.000	0.128	0.000	0.159	2741102	125702	4963339	462288	637065	61565	786939	48649

389.177	14.5	Asn-Gln-Gln	0.009	21.868	0.299	0.711	0.541	0.823	4402	487	96268	21970	68490	11732	79220	15394
389.304	3.8	[PR (2:0)] (+)-24,25-epoxy-16-scalaren-12alpha,25alpha-diol	0.000	4.022	0.000	0.199	0.000	0.231	830741	68662	3341319	289466	666368	62098	772931	49533
391.320	3.8	[ST (3:0/3:0)] (5Z,7E)-A-dinor-(1,2)-(9,10)-diseco-5,7,10(19)-cholestatriene-1,2,25-triol	0.000	8.051	0.000	0.190	0.000	0.160	61423	6330	494507	33485	93841	13803	79354	20059
395.160	15.0	Ala-Met-Ser-Ser	0.043	#DIV/0!	0.005	24.910	0.005	15.895	0	0	3361	1247	83732	16740	53430	10637
396.018	14.2	Molybdopterin	0.000	2.819	0.041	1.399	0.118	1.316	103183	5189	290831	11181	406809	42743	382817	48495
399.144	16.5	Deoxypodophyllotoxin	0.001	12.274	0.002	2.272	0.001	3.429	236785	14354	2906365	422510	6604120	719397	9967214	1194973
400.147	16.6	Glu-Asp-His	0.002	25.843	0.003	2.298	0.001	3.502	16218	4560	419119	65210	963253	108646	1467753	178492
400.341	4.7	[FA] O-Palmitoyl-R-carnitine	0.000	3.586	0.005	1.653	0.783	0.965	1712161	87193	6139616	562898	10148843	885056	5926741	499288
400.342	7.5	[FA] O-Palmitoyl-R-carnitine	0.001	3.596	0.005	1.758	0.619	0.929	183702	9915	660542	71635	1161363	112308	613720	56352
401.140	16.5	[Fv methoxy,methox] (S)-2,3-Dihydro-7-methoxy-2-[2-(4-methoxyphenyl)-5-benzofuranyl]-4H-1-benzopyran-4-one	0.003	121.893	0.002	2.657	0.002	4.021	799	362	97430	18215	258825	30394	391809	53346
402.143	16.6	Met-Asp-His	0.006	#DIV/0!	0.027	5.245	0.011	9.886	0	0	3307	721	17345	4564	32696	7551
402.144	12.5	Met-Asp-His	0.002	10.007	0.000	9.927	0.000	11.976	1049	337	10501	1653	104241	8169	125762	9594
405.009	18.9	UDP	0.049	1.786	0.504	1.192	0.028	1.832	9383	1636	16758	2740	19979	3731	30693	4457
405.208	14.8	Asn-Lys-Gly-Ser	0.001	0.158	0.002	9.879	0.006	11.343	47575	6081	7518	2160	74270	11134	85278	17247
407.135	14.1	Astringin	0.001	65.156	0.518	0.833	0.392	1.243	481	305	31372	4163	26135	6544	39006	7329
409.366	3.9	[FA oxo(26:0)] 17-oxo-20Z-hexacosenoic acid	0.042	0.581	0.240	1.278	0.327	1.367	20168	3111	11716	1218	14977	2254	16020	3849
411.346	7.4	MG(0:0/22:2(13Z,16Z)/0:0)	0.054	0.236	0.730	1.366	0.970	1.037	944024	284081	222987	127590	304640	190041	231235	167945
413.362	3.9	MG(0:0/22:1(13Z)/0:0)	0.034	0.342	0.681	1.252	0.531	1.429	80285	18394	27464	7679	34382	14256	39235	16140
414.320	4.9	3-Hydroxy-9-hexadecenoylcarnitine	0.002	29.177	0.014	2.715	0.000	4.851	1707	782	49818	8638	135242	23695	241672	22735
414.357	4.6	Heptadecanoylcarnitine	0.000	5.873	0.000	2.262	0.132	1.294	57517	9759	337774	34993	764109	60847	437192	48796
424.341	4.6	Linoelaidylcarnitine	0.000	3.512	0.107	1.701	0.169	0.796	23441	3670	82335	7426	140015	29197	65532	8523
425.043	14.9	Thiamin diphosphate	0.019	2.807	0.861	1.059	0.010	1.861	38319	5412	107578	20539	113921	28514	200255	21115
426.357	4.6	Elaidiccarnitine	0.000	5.244	0.009	1.587	0.978	1.003	528837	26253	2773251	250965	4402310	412106	2782716	216033
428.036	14.2	ADP	0.002	2.272	0.128	1.246	0.024	1.425	195741	9463	444776	41888	553991	50487	633670	56047
428.036	15.2	ADP	0.001	3.914	0.433	1.119	0.055	1.326	187555	16200	734028	82191	821350	68021	973369	73468
428.036	16.6	ADP	0.002	3.752	0.004	3.800	0.003	4.440	2568	431	9637	1265	36615	5543	42783	6206
428.373	4.6	Stearoylcarnitine	0.005	1.898	0.001	2.351	0.003	1.699	600357	27947	1139440	116577	2678949	264670	1935857	161004
429.088	4.0	Diethylstilbestrol diphosphate	0.042	0.284	0.545	1.384	0.318	16.093	33127	8788	9419	2995	13031	4875	151571	128133
429.239	16.8	Aspidoalbine	0.043	3.784	0.064	0.351	0.031	0.186	642831	157442	2432774	665700	854830	158342	452913	83270
430.243	17.7	Lys-Trp-Pro	0.003	6.351	0.005	0.184	0.003	0.111	118987	38869	755667	129762	138761	26406	84241	11939
431.237	18.4	Gln-Gln-Arg	0.018	0.610	0.014	1.607	0.339	1.303	26094	2658	15930	2400	25592	2160	20761	4099
433.330	9.3	[ST (2:0)] (7E)-(1S,3R,6R)-6,19-epidioxy-9,10-seco-5(10),7-cholestadiene-1,3-diol	0.011	2.245	0.298	0.721	0.120	0.705	21246	2314	47706	6919	34387	9881	33620	4260

438.297	4.7	[PE (16:1)] 1-(1Z-hexadecenyl)-sn-glycero-3-phosphoethanolamine	0.001	2.876	0.048	0.756	0.524	0.928	500958	16723	1440707	133247	1088567	64701	1336956	81029
438.297	7.6	[PE (16:1)] 1-(1Z-hexadecenyl)-sn-glycero-3-phosphoethanolamine	0.001	3.098	0.036	0.716	0.121	0.803	86121	4557	266793	25831	191049	15863	214128	16276
441.297	13.5	[ST (2:0/2:0/2:0)] (5Z,7E)-(3S)-26,26,26-trifluoro-27-nor-9,10-seco-5,7,10(19)-cholestatriene-3,25-diol	0.000	2.046	0.418	1.189	0.220	1.201	11852028	1427059	24251208	393787	28839186	5190261	29134598	3474263
441.297	14.4	[ST (2:0/2:0/2:0)] (5Z,7E)-(3S)-26,26,26-trifluoro-27-nor-9,10-seco-5,7,10(19)-cholestatriene-3,25-diol	0.000	0.090	0.134	4.069	0.442	1.193	45897171	612440	4141756	736907	16852024	7097592	4940125	670798
441.393	3.9	MG(0:0/24:1(15Z)/0:0)	0.024	0.276	0.368	1.619	0.928	1.034	102418	23677	28281	8637	45790	16130	29248	5666
442.352	7.6	3-Hydroxy-11Z-octadecenoylcarnitine	0.001	#DIV/0!	0.000	13.029	0.000	11.175	0	0	17797	2668	231870	23267	198872	21113
444.031	18.1	GDP	0.008	13.538	0.733	0.896	0.489	1.200	3981	945	53892	11925	48287	10635	64661	8979
444.031	17.5	GDP	0.000	11.759	0.002	2.320	0.012	1.579	4602	609	54110	4717	125559	13066	85423	8438
447.067	16.3	CDP-ethanolamine	0.000	27.687	0.064	1.373	0.004	1.915	1601	504	44339	4155	60887	6546	84894	8865
448.149	15.7	Asn-Asp-Cys-Pro	0.000	#DIV/0!	0.000	0.000	0.000	0.042	0	0	74319	8740	0	0	3103	976
452.312	4.7	[PC (14:1)] 1-(1E-tetradecenyl)-sn-glycero-3-phosphocholine	0.004	3.832	0.012	0.429	0.058	0.594	51626	9802	197844	30602	84814	14535	117549	20236
454.292	14.5	[PE (16:0)] 1-hexadecanoyl-sn-glycero-3-phosphoethanolamine	0.000	0.163	0.081	3.601	0.846	0.947	730525	31725	119222	21284	429298	142273	112872	23683
460.193	14.2	5-Methyltetrahydrofolate	0.050	#DIV/0!	0.013	3.039	0.004	4.201	0	0	4073	1579	12379	2205	17110	2891
465.176	18.8	Enhydrin	0.000	#DIV/0!	0.000	0.000	0.000	0.000	0	0	115644	9504	0	0	0	0
466.328	4.6	[PC (15:1)] 1-(1Z-pentadecenyl)-sn-glycero-3-phosphocholine	0.001	2.346	0.004	0.627	0.023	0.742	623617	24153	1462770	116877	916800	63406	1085071	63010
466.328	7.5	[PC (15:1)] 1-(1Z-pentadecenyl)-sn-glycero-3-phosphocholine	0.005	2.601	0.028	0.599	0.072	0.693	82432	1661	214412	28094	128456	14509	148635	13604
467.332	4.6	[ST (3:0/3:0/3:0)] (5Z,7E)-(1S,3R)-24,24-difluoro-24a-homo-9,10-seco-5,7,10(19)-cholestatrien-1,3,25-triol	0.006	2.677	0.034	0.558	0.051	0.613	113667	17714	304334	44232	169721	30257	186511	26573
473.119	14.4	[FA methyl,oxo,hydroxy(4:0)] methyl 9-oxo-10-iodo-12R-hydroxy-5Z,7E,10Z,13Z-prostatetraenoate-cyclo[8,12]	0.001	14.970	0.001	0.173	0.001	0.147	15241	2063	228169	27273	39585	7335	33437	5391
473.319	15.9	Ala-Lys-Val-Arg	0.030	0.045	0.000	25.047	0.001	23.576	443487	140774	19867	4126	497615	37505	468390	69929
478.328	4.7	[PC (16:2)] 1-(9E,10E-hexadecadienyl)-sn-glycero-3-phosphocholine	0.015	2.649	0.067	2.657	0.003	4.121	9712	1526	25732	4533	68362	18296	106056	15623
480.308	4.7	[PE (18:0)] 1-(9Z-octadecenoyl)-sn-glycero-3-phosphoethanolamine	0.000	5.449	0.181	1.167	0.029	1.298	105689	6215	575860	50872	672011	43240	747576	44065
480.344	4.7	[PC (16:1)] 1-(1Z-hexadecenyl)-sn-glycero-3-phosphocholine	0.006	1.894	0.013	1.510	0.006	1.552	382646	20452	724573	77458	1093920	94143	1124878	82957
481.313	3.9	His-Leu-Leu-Val	0.043	0.105	0.252	1.540	0.090	1.745	66309	21962	6978	1345	10746	2716	12175	2333
482.323	4.7	[PC (15:0)] 1-pentadecanoyl-sn-glycero-3-phosphocholine	0.002	1.734	0.746	0.951	0.637	0.932	2496900	263931	4330205	357993	4116063	530253	4036747	482354
482.323	14.5	[PC (15:0)] 1-pentadecanoyl-sn-glycero-3-phosphocholine	0.000	0.217	0.000	5.554	0.118	2.908	861053	60878	187130	20567	1039228	25880	544197	188804
482.323	8.2	[PC (15:0)] 1-pentadecanoyl-sn-glycero-3-phosphocholine	0.027	1.984	0.314	0.788	0.630	0.871	36621	7708	72674	11283	57255	9106	63327	14967
482.323	7.5	[PC (15:0)] 1-pentadecanoyl-sn-glycero-3-phosphocholine	0.019	2.036	0.033	0.632	0.197	0.723	924676	288614	1882385	133127	1189677	232689	1360405	338288
482.360	4.8	[PC (16:2)] 1-hexadecyl-sn-glycero-3-phosphocholine	0.005	0.637	0.000	3.288	0.000	3.613	1184766	67179	754294	94292	2480094	222446	2725040	245983
483.991	18.4	CTP	0.009	12.294	0.753	1.090	0.064	1.639	8171	1900	100455	22160	109475	16854	164607	21489

484.975	17.9	UTP	0.001	0.534	0.000	7.534	0.000	11.550	93098	4825	49715	7288	374553	38565	574215	54969
487.310	7.5	Arg-Val-Gly-Arg	0.046	0.258	0.910	0.972	0.079	5.754	986016	277424	254076	24970	246948	55087	1461842	549285
489.114	15.3	CDP-choline	0.001	22.575	0.001	0.142	0.001	0.191	34937	4543	788678	97743	111835	16102	150416	18829
494.323	7.6	[PC (16:0)] 1-(9Z-hexadecenoyl)-sn-glycero-3-phosphocholine	0.001	9.538	0.001	5.373	0.000	5.249	5320	1894	50737	6470	272614	31964	266323	26607
498.219	15.6	Validamycin A	0.012	6.196	0.008	0.066	0.015	0.224	5532	1282	34278	7555	2252	903	7685	2303
504.306	4.6	LysoPE(0:0/20:3(11Z,14Z,17Z))	0.000	7.169	0.298	1.099	0.780	1.039	46609	6898	334141	22230	367386	20583	347271	39519
506.360	4.7	[PC (18:2)] 1-(1Z,9Z-octadecadienyl)-sn-glycero-3-phosphocholine	0.006	4.027	0.766	0.932	0.808	1.055	36910	5169	148624	24999	138461	21896	156839	21340
507.201	8.1	Piperaduncin B	0.049	#DIV/0!	0.051	0.015	0.055	0.042	0	0	43259	16672	630	400	1799	447
507.201	4.9	Piperaduncin B	0.028	#DIV/0!	0.032	0.036	0.057	0.196	0	0	79675	26031	2830	1319	15606	3966
508.002	16.6	ATP	0.002	1.987	0.001	2.006	0.001	2.416	1689875	77228	3357241	295662	6734471	590667	8111765	717997
508.338	4.7	[PC (17:0)] 1-(10Z-heptadecenoyl)-sn-glycero-3-phosphocholine	0.013	8.581	0.000	5.318	0.000	6.127	14419	4972	123735	29509	658053	56566	758087	59018
508.375	4.7	[PC (18:1)] 1-(11Z-octadecenyl)-sn-glycero-3-phosphocholine	0.037	1.540	0.013	1.558	0.006	1.622	719819	31856	1108816	138592	1727458	149434	1798251	145714
510.305	27.3	Gln-Leu-Leu-His	0.052	0.610	0.347	1.229	0.025	1.449	66533	9885	40590	5476	49871	7583	58797	4066
510.355	7.6	LysoPC(17:0)	0.041	1.479	0.001	4.908	0.001	5.607	30581	2257	45240	5348	222056	25263	253654	30015
516.300	7.5	Taurocholate	0.026	0.428	0.236	1.754	0.254	1.738	1746590	297148	747904	235511	1312043	374347	1299547	383938
519.324	4.8	Hodgkinsine	0.028	1.557	0.000	4.214	0.000	5.182	179177	12406	278983	33025	1175670	75465	1445672	89269
520.340	4.7	[PC (18:2)] 1-(9Z,12Z-octadecadienoyl)-sn-glycero-3-phosphocholine	0.005	52.061	0.000	4.666	0.000	5.703	3723	2548	193819	39395	904396	93779	1105255	112814
521.343	4.7	Lys-Lys-Phe-Val	0.005	57.756	0.001	4.975	0.002	7.021	782	782	45173	9512	224736	27608	317142	46948
522.354	4.7	1-Oleoylglycerophosphocholine	0.003	4.140	0.000	4.038	0.000	4.998	1050702	64333	4349473	604148	17562470	1456642	21738895	1803978
522.355	7.5	1-Oleoylglycerophosphocholine	0.002	3.996	0.000	4.799	0.000	5.347	70931	4446	283429	35197	1360049	125318	1515550	158314
523.997	19.3	GTP	0.000	7.584	0.060	1.485	0.002	2.214	23676	6051	179560	13191	266656	35662	397535	40427
526.292	4.6	LysoPE(0:0/22:6(4Z,7Z,10Z,13Z,16Z,19Z))	0.001	2.359	0.214	0.853	0.136	0.813	201254	14089	474664	42078	405074	30659	385937	34693
528.308	4.6	LysoPE(0:0/22:5(4Z,7Z,10Z,13Z,16Z))	0.000	3.192	0.190	0.877	0.032	0.728	129104	16146	412101	26969	361325	23894	300211	35297
529.311	4.6	Glu-Lys-Pro-Arg	0.015	2.814	0.825	1.067	0.317	0.725	26891	4499	75675	13898	80743	17352	54864	14047
536.370	4.7	[PC (16:2/3:0)] 1-hexadecyl-2-(2E-propionyl)-sn-glycero-3-phosphocholine	0.006	5.406	0.000	4.113	0.000	5.540	16248	3175	87835	16147	361274	31621	486638	38912
538.386	4.6	[PC acetyl(17:2)] 1-heptadecyl-2-acetyl-sn-glycero-3-phosphocholine	0.002	0.383	0.000	11.862	0.000	12.722	139172	15723	53328	8042	632577	51818	678458	55359
542.067	14.2	Cyclic ADP-ribose	0.004	2.225	0.136	1.278	0.028	1.475	142675	6598	317407	34809	405544	41527	468155	46433
543.300	14.9	Arg-Asp-Pro-Arg	0.019	0.481	0.168	1.750	0.274	6.172	62416	7052	30031	8997	52557	12065	185337	126178
545.341	4.7	Glu-Leu-Lys-Arg	0.021	1.668	0.000	2.782	0.000	3.283	194299	9606	324055	39681	901482	67857	1063936	70643
548.373	4.6	LysoPC(20:2(11Z,14Z))	0.000	#DIV/0!	0.000	12.825	0.001	19.504	0	0	14487	1271	185785	18099	282549	33704
550.386	4.6	LysoPC(20:1(11Z))	0.001	3.159	0.000	4.528	0.000	6.146	68282	8304	215702	22813	976611	77461	1325598	109050
552.402	4.6	[PC (20:0)] 1-eicosanoyl-sn-glycero-3-phosphocholine	0.004	0.498	0.000	8.458	0.000	10.813	135444	6410	67426	14580	570294	44001	729094	50835

568.339	4.6	[PC (22:6)] 1-(4Z,7Z,10Z,13Z,16Z,19Z-docosahexaenoyl)-sn-glycero-3-phosphocholine	0.009	2.501	0.000	3.014	0.000	2.920	177353	15799	443606	66533	1336821	113711	1295128	112524
570.354	4.6	LysoPC(22:5(4Z,7Z,10Z,13Z,16Z))	0.018	2.029	0.000	3.403	0.000	3.515	146497	15303	297302	44321	1011806	90392	1044876	99060
574.093	12.6	GDP-3,6-dideoxy-D-galactose	0.001	#DIV/0!	0.001	0.046	0.007	0.346	0	0	24071	3708	1114	501	8329	2517
594.184	17.5	[Fv] Tinctormine	0.026	2.428	0.038	0.470	0.935	0.976	6307	995	15311	2920	7194	1043	14941	3326
594.310	17.5	Integerrine	0.015	2.058	0.024	0.544	0.411	0.826	9913	1617	20404	2954	11100	1412	16855	2899
603.534	3.9	[GL methyl(15:0/8:0)] 1-(14-methyl-pentadecanoyl)-2-(8-[3]-ladderane-octanyl)-sn-glycerol	0.000	4.919	0.070	1.255	0.031	1.257	643542	20296	3165874	75048	3972260	350184	3978380	276769
606.448	4.5	LysoPC(24:1(15Z))	0.043	1.759	0.003	4.946	0.000	16.524	18969	2607	33361	5281	164997	25511	551278	40693
613.159	17.4	Glutathione disulfide	0.001	5.952	0.000	24.600	0.000	20.853	60336	6792	359117	47498	8834215	908168	7488535	730053
615.152	17.4	CMP-N-acetylneuraminate	0.007	#DIV/0!	0.000	37.906	0.000	32.277	0	0	14698	3314	557132	53830	474395	48779
615.171	17.4	[Fv] Safflomin C	0.026	#DIV/0!	0.000	112.807	0.000	90.846	0	0	1793	571	202306	21845	162921	17263
621.447	17.3	[GP (15:0/15:0)] 1,2-dipentadecanoyl-sn-glycero-3-phosphate	0.006	0.024	0.087	73.764	0.635	0.834	952963	203685	22614	6753	1668092	774996	18859	3444
622.070	17.4	UDP-N-acetyl-D-mannosaminouronate	0.018	11.178	0.322	0.678	0.531	1.212	14887	3742	166397	44121	112834	24863	201635	31256
631.352	9.9	Remikiren	0.046	0.651	0.266	2.991	0.289	1.308	19220	1969	12504	2179	37401	19839	16360	2657
635.141	17.4	Actinorhodine	0.002	13.677	0.000	33.987	0.000	29.620	5153	864	70478	11307	2395342	287574	2087547	226820
651.533	3.8	[GL (8:0/8:0)] 1-(8-[3]-ladderane-octanoyl-2-(8-[3]-ladderane-octanyl)-sn-glycerol	0.000	4.042	0.000	0.380	0.000	0.502	473039	17563	1911824	95290	725889	63628	958878	65655
664.115	14.2	NAD+	0.002	2.187	0.110	1.314	0.019	1.528	876648	39348	1916909	182379	2518756	283432	2929384	297045
666.131	13.3	NADH	0.009	2.221	0.001	2.477	0.001	3.351	32381	2657	71924	9816	178126	17264	241025	28750
672.539	3.8	Galactosylceramide (d18:1/14:0)	0.051	0.399	0.385	0.704	0.528	0.775	145221	33754	57940	15163	40802	11095	44931	12833
675.542	7.5	[FA (32:0/2:0)] 1-(O-alpha-D-glucopyranosyl)-29-keto-(3R,31R)-dotriacontanediol	0.054	2.152	0.068	0.484	0.051	0.454	188302	10002	405147	86533	195999	47079	183878	30004
675.542	4.4	[FA (32:0/2:0)] 1-(O-alpha-D-glucopyranosyl)-29-keto-(3R,31R)-dotriacontanediol	0.004	1.698	0.006	0.633	0.008	0.646	2398842	91188	4073426	354251	2580132	180099	2631100	191001
676.494	17.4	PC(14:0/14:1(9Z))	0.001	15.780	0.138	0.640	0.929	1.028	5091	1235	80328	11534	51398	13708	82553	21354
678.505	4.2	[PC (14:0/14:0)] 1,2-ditetradecanoyl-sn-glycero-3-phosphocholine	0.010	56.793	0.000	5.810	0.000	11.158	6262	2039	355632	85688	2066192	185708	3968213	314615
681.212	17.3	Canaliculatol	0.000	4.754	0.304	0.831	0.337	1.166	64944	11733	308742	32297	256485	35700	359991	39079
681.641	17.3	[GL (20:0/20:0)] 1,2-dieicosanoyl-sn-glycerol	0.005	3.209	0.266	0.765	0.503	1.332	8618	363	27657	3917	21168	3867	36827	12265
689.557	4.4	[SP (18:0/14:0)] N-(octadecanoyl)-tetradecasphing-4-enine-1-phosphoethanolamine	0.004	1.607	0.001	0.506	0.001	0.505	2514053	90205	4039779	310469	2044312	166892	2041816	131845
690.506	4.2	PC(14:1(9Z)/15:0)	0.000	17.875	0.030	2.626	0.026	5.770	3371	1263	60256	6622	158218	32988	347674	91491
692.521	4.2	[PE (16:0/16:0)] 1,2-dihexadecanoyl-sn-glycero-3-phosphoethanolamine	0.001	41.032	0.001	2.385	0.000	2.870	21862	3475	897058	125752	2139431	196652	2574357	231475
692.557	4.2	[PC (14:2/16:0)] 1-tetradecyl-2-hexadecanoyl-sn-glycero-3-phosphocholine	0.003	3.262	0.037	1.510	0.375	1.208	351422	58222	1146455	159898	1731542	183013	1384612	199693
700.526	4.1	PE(16:1(9Z)/P-18:1(11Z))	0.001	8.954	0.004	0.401	0.002	0.321	17057	4368	152735	19955	61278	13078	48996	9033

702.506	4.2	PC(14:1(9Z)/16:1(9Z))	0.003	#DIV/0!	0.004	6.014	0.000	20.558	0	0	30908	5797	185873	31460	635420	65514
702.542	4.1	PE(16:0/P-18:1(11Z))	0.034	2.856	0.702	0.900	0.054	0.433	237640	41978	678628	153425	610626	74284	293747	34102
703.573	7.5	[SP (16:0)] N-(hexadecanoyl)-sphing-4-enine-1-phosphocholine	0.006	2.054	0.003	0.429	0.001	0.345	5053328	204855	10380827	1177299	4449733	934464	3585745	644499
703.573	4.4	[SP (16:0)] N-(hexadecanoyl)-sphing-4-enine-1-phosphocholine	0.001	2.107	0.004	0.601	0.001	0.544	42348986	1726884	89245086	7223464	53605724	6223221	48552418	3836350
704.521	4.2	[PC (14:0/16:1)] 1-tetradecanoyl-2-(9Z-hexadecenoyl)-sn-glycero-3-phosphocholine	0.000	22.946	0.001	1.989	0.000	2.762	167130	43630	3834927	360086	7626626	622404	10591371	750361
705.591	4.3	[SP (16:0)] N-(hexadecanoyl)-sphinganine-1-phosphocholine	0.053	19.095	0.411	1.491	0.097	2.204	4177	4177	79768	29960	118951	34393	175784	42284
706.537	4.2	[PC (15:0/15:0)] 1,2-dipentadecanoyl-sn-glycero-3-phosphocholine	0.000	7.943	0.642	1.058	0.303	1.113	3248206	144200	25799966	1762342	27303941	2578211	28714790	2022593
706.573	4.2	[PE (18:2/16:0)] 1-octadecyl-2-hexadecanoyl-sn-glycero-3-phosphoethanolamine	0.000	0.222	0.049	3.313	0.123	1.622	400172	27403	88726	15179	293968	79264	143949	28059
707.577	4.2	2-demethylmenaquinol-8	0.007	0.337	0.312	1.416	0.924	0.966	155552	25191	52363	13908	74148	14995	50586	11477
708.543	4.2	[PR] bacteriohopane-,32,33,34-triol-35-cyclitol	0.000	21.231	0.689	1.046	0.239	1.127	113639	9942	2412673	161584	2523766	214804	2718506	182584
716.521	4.2	[PE (16:0/18:2)] 1-hexadecanoyl-2-(9Z,12Z-octadecadienoyl)-sn-glycero-3-phosphoethanolamine	0.000	123.821	0.008	0.610	0.033	0.832	9330	2471	1155220	64920	704755	111818	961165	38921
716.557	4.2	PC(14:0/P-18:1(11Z))	0.000	3.155	0.095	1.240	0.154	1.185	376779	58714	1188675	92056	1473391	122487	1408860	108810
717.552	4.4	SM(d18:0/16:1(9Z)(OH))	0.036	0.590	0.837	0.931	0.341	0.732	163778	11471	96629	23419	89957	21264	70705	9554
718.537	4.2	[PE (16:0/18:1)] 1-Hexadecanoyl-2-(9Z-octadecenoyl)-sn-glycero-3-phosphoethanolamine	0.000	8.686	0.693	0.960	0.485	1.072	983473	44824	8542559	533192	8197762	657242	9155691	655536
719.577	4.2	menaquinol-8	0.000	3.304	0.095	1.244	0.064	1.246	1310720	79700	4330688	304262	5387138	473117	5396195	406574
720.553	4.2	[PC (15:0/16:0)] 1-pentadecanoyl-2-hexadecanoyl-sn-glycero-3-phosphocholine	0.000	3.186	0.700	0.956	0.331	0.906	3914685	201207	12471734	734632	11919115	1175906	11304723	873649
720.590	4.2	[PC (14:2/18:0)] 1-tetradecyl-2-octadecanoyl-sn-glycero-3-phosphocholine	0.002	0.593	0.058	1.474	0.280	0.821	4571089	323305	2710422	78022	3995189	525394	2225289	396269
721.593	4.2	dihydromenaquinone-8	0.000	0.365	0.004	2.453	0.534	0.772	1953910	153408	713431	172698	1749933	213214	550577	184914
722.511	4.1	PE(18:4(6Z,9Z,12Z,15Z)/P-18:1(11Z))	0.000	9.197	0.000	0.096	0.000	0.165	135896	63482	1249796	97051	120291	27181	206189	26952
726.542	4.1	PE(18:2(9Z,12Z)/P-18:1(11Z))	0.001	14.865	0.800	0.918	0.043	0.512	17817	11502	264836	41271	243138	72004	135596	37340
728.558	4.1	PE(18:1(11Z)/P-18:1(11Z))	0.004	3.845	0.652	0.872	0.025	0.507	53175	8937	204459	31140	178268	46597	103588	20535
728.602	3.8	Glucosylceramide (d18:1/18:0)	0.035	0.397	0.411	1.382	0.954	0.974	257735	52088	102307	33430	141407	30868	99624	31015
730.537	4.2	[PC (14:0/18:2)] 1-tetradecanoyl-2-(9Z,12Z-octadecadienoyl)-sn-glycero-3-phosphocholine	0.000	5.214	0.388	1.103	0.011	1.400	1732284	88513	9031782	739091	9958553	711873	12640801	892296
730.573	4.5	[PE (18:1/18:1)] 1-(1Z-octadecenyl)-2-(9Z-octadecenoyl)-sn-glycero-3-phosphoethanolamine	0.044	3.864	0.153	0.512	0.060	0.330	22887	5720	88434	24531	45310	11048	29204	4384
732.552	4.2	[PC (14:0/18:1)] 1-tetradecanoyl-2-(11Z-octadecenoyl)-sn-glycero-3-phosphocholine	0.000	4.953	0.423	0.949	0.322	1.086	20800325	853056	103016197	5424394	97786846	2976357	111886325	6532772
734.495	4.0	PS(14:0/18:1(9Z))	0.000	3.222	0.302	1.390	0.036	1.919	12369	2350	39848	4038	55387	13125	76450	12941

740.521	4.1	[PE (16:0/20:4)] 1-hexadecanoyl-2-(5Z,8Z,11Z,14Z-eicosatetraenoyl)-sn-glycero-3-phosphoethanolamine	0.000	8.352	0.000	0.419	0.000	0.474	349528	59015	2919247	171382	1224012	86311	1384005	51506
742.535	4.2	[PE (18:1/18:2)] 1-(9Z-octadecenoyl)-2-(9Z,12Z-octadecadienoyl)-sn-glycero-3-phosphoethanolamine	0.000	11.675	0.001	0.467	0.000	0.526	182300	65763	2128357	110946	993114	206357	1119172	38923
742.573	4.2	[PC (16:1/18:2)] 1-(1Z-hexadecenyl)-2-(9Z,12Z-octadecadienoyl)-sn-glycero-3-phosphocholine	0.000	10.741	0.007	0.429	0.044	0.716	91081	20203	978298	57613	420077	133060	700773	100800
744.081	16.7	NADP+	0.001	5.057	0.205	1.253	0.039	1.596	20437	5280	103348	13126	129451	14071	164970	21510
744.496	20.2	Pilocereine	0.024	8.827	0.228	0.576	0.066	0.351	4311	1004	38055	10556	21901	6503	13346	2140
744.552	4.2	[PE (18:0/18:2)] 1-octadecanoyl-2-(9Z,12Z-octadecadienoyl)-sn-glycero-3-phosphoethanolamine	0.000	13.342	0.001	0.619	0.001	0.640	742900	40755	9911590	618038	6138855	478149	6339678	422913
744.589	4.2	1-Hexadecanoyl-2-(9Z-octadecenoyl)-sn-glycero-3-phosphonocholine	0.000	5.822	0.040	0.754	0.070	0.791	1675867	124341	9757366	773989	7360630	648908	7718068	637451
746.0967	17.0196	NADPH	0.087	1.316	0.149	0.740	0.062	0.678	63155	3091	83107	9276	61481	10264	56387	8686
746.568	4.2	[PC (15:0/18:1)] 1-pentadecanoyl-2-(11Z-octadecenoyl)-sn-glycero-3-phosphocholine	0.000	5.821	0.054	0.792	0.153	0.858	5891914	251477	34297978	2388390	27151174	2225186	29437569	2027397
746.604	4.2	PC(16:0/P-18:0)	0.007	2.587	0.453	1.125	0.617	1.080	9195500	439308	23785057	3358041	26750554	1640514	25684474	1342967
748.526	4.1	[PE (16:1/22:6)] 1-O-(1Z-hexadecenyl)-2-(4Z,7Z,10Z,13Z,16Z,19Z-docosahexaenoyl)-sn-glycero-3-phosphoethanolamine	0.000	2.193	0.001	0.541	0.000	0.273	4595668	216668	10077830	593266	5452707	695015	2753189	253071
748.585	4.2	[PE (16:0/20:0)] 1-hexadecanoyl-2-eicosanoyl-sn-glycero-3-phosphoethanolamine	0.000	1.842	0.008	0.370	0.000	0.258	2768995	197065	5101436	226246	1885504	780613	1318326	547528
750.542	4.1	PE(20:4(5Z,8Z,11Z,14Z)/P-18:1(11Z))	0.000	1.706	0.000	0.537	0.000	0.459	8941490	420174	15253332	821377	8185203	816201	7002431	549023
756.552	4.2	[PC (16:0/18:3)] 1-hexadecanoyl-2-(9Z,12Z,15Z-octadecatrienoyl)-sn-glycero-3-phosphocholine	0.001	2.343	0.947	0.993	0.134	1.182	3376899	154241	7912806	627936	7856270	542171	9352365	620072
756.633	3.8	Glucosylceramide (d18:1/20:0)	0.022	0.223	0.025	2.916	0.202	1.641	218694	52082	48707	7961	142030	29945	79919	20472
758.568	4.2	[PC (16:0/18:2)] 1-hexadecanoyl-2-(9Z,12Z-octadecadienoyl)-sn-glycero-3-phosphocholine	0.000	7.299	0.235	0.870	0.973	1.004	11197575	471243	81734280	6577733	71128217	5161690	82042963	5942018
760.511	3.9	PS(16:0/18:2(9Z,12Z))	0.000	8.615	0.000	0.222	0.000	0.352	35511	5802	305912	23256	67840	16963	107730	9360
762.526	3.9	[PS (16:0/18:1)] 1-hexadecanoyl-2-(9Z-octadecenoyl)-sn-glycero-3-phosphoserine	0.000	2.555	0.000	0.299	0.000	0.317	1478723	89766	3777682	122591	1131148	129363	1197922	91231
762.599	4.1	[PC (18:0/16:0)] 1-octadecanoyl-2-hexadecanoyl-sn-glycero-3-phosphocholine	0.004	0.000	0.194	0.000	0.194	0.000	9459287	1908879	2982	1991	0	0	0	0
764.522	4.1	[PE (16:0/22:6)] 1-hexadecanoyl-2-(4Z,7Z,10Z,13Z,16Z,19Z-docosahexaenoyl)-sn-glycero-3-phosphoethanolamine	0.000	14.864	0.000	0.326	0.000	0.400	108185	27214	1608061	61891	524737	125974	643544	117077
766.537	4.1	PE(20:2(11Z,14Z)/18:3(6Z,9Z,12Z))	0.000	25.532	0.000	0.375	0.000	0.447	223884	73861	5716219	393694	2142891	182417	2553929	151750
766.572	4.2	[PC (P-16:0/20:4)] 1-(1Z-hexadecenyl)-2-(5Z,8Z,11Z,14Z-eicosatetraenoyl)-sn-glycero-3-phosphocholine	0.000	0.558	0.343	0.896	0.031	0.749	11030694	558606	6154030	504544	5515277	392407	4607039	327823
768.552	4.1	[PE (18:0/20:4)] 1-octadecanoyl-2-(5Z,8Z,11Z,14Z-eicosatetraenoyl)-sn-glycero-3-phosphoethanolamine	0.000	2.425	0.000	0.524	0.000	0.484	7225857	333852	17519672	927223	9173026	858235	8476912	609435

768.588	4.2	PC(18:2(9Z,12Z)/P-18:1(11Z))	0.000	0.485	0.370	0.901	0.046	0.771	38838385	1729237	18842757	1521488	16974615	1281000	14529328	1086263
770.569	4.2	PC(15:0/20:3(5Z,8Z,11Z))	0.002	320.017	0.068	0.586	0.082	0.643	12928	6835	4137306	679520	2422511	468632	2661585	115112
770.605	4.2	PC(18:1(11Z)/P-18:1(11Z))	0.000	14855.241	0.002	0.586	0.001	0.561	487	487	7238151	556683	4238317	383678	4059904	322527
771.608	4.2	demethylmenaquinone-9	0.000	47.594	0.002	0.583	0.001	0.537	76064	9769	3620179	281639	2110348	187214	1942530	149634
772.583	4.2	[PE (18:0/20:2)] 1-octadecanoyl-2-(11Z,14Z-eicosadienoyl)-sn-glycero-3-phosphoethanolamine	0.000	14.308	0.014	0.710	0.104	0.823	1039623	141329	14874836	1147340	10568295	818233	12246527	903031
772.620	4.2	[PC (18:1/18:0)] 1-(1Z-octadecenyl)-2-(9Z-octadecenoyl)-sn-glycero-3-phosphocholine	0.000	8.554	0.007	0.684	0.006	0.693	1103691	139694	9441109	661744	6456750	563116	6539003	496351
773.623	4.2	demethylmenaquinol-9	0.000	15.757	0.004	0.666	0.003	0.673	289740	78512	4565304	301518	3042497	287004	3072861	225753
774.599	4.2	[PE (16:0/22:1)] 1-hexadecanoyl-2-(13Z-docosenoyl)-sn-glycero-3-phosphoethanolamine	0.000	5.537	0.010	0.715	0.002	0.667	4205574	198156	23284272	1384028	16647679	1553280	15541641	1177268
774.636	4.2	PC(18:0/P-18:0)	0.007	3.905	0.024	7.519	0.265	2.747	32598	7740	127304	22759	957254	261071	349756	176551
776.557	4.0	[PE (18:1/22:6)] 1-(1Z-octadecenyl)-2-(4Z,7Z,10Z,13Z,16Z,19Z-docosahexaenoyl)-sn-glycero-3-phosphoethanolamine	0.000	2.010	0.000	0.197	0.000	0.156	2624580	154618	5276609	209759	1040554	202076	820785	125267
777.561	4.0	[PG (18:0/18:1)] 1-octadecanoyl-2-(9Z-octadecenoyl)-sn-glycero-3-phospho-(1'-sn-glycerol)	0.005	1.762	0.000	0.307	0.000	0.205	1267834	63902	2233470	210503	685699	159906	458844	46922
779.608	21.7	Decaprenol phosphate	0.012	3.212	0.068	3.003	0.016	4.941	16970	4328	54510	10106	163717	47078	269312	60420
780.551	4.2	[PC (16:1/20:4)] 1-(9Z-hexadecenoyl)-2-(5Z,8Z,11Z,14Z-eicosatetraenoyl)-sn-glycero-3-phosphocholine	0.001	2.335	0.275	0.886	0.750	1.035	5092454	272206	11890658	961857	10538159	651678	12310547	845960
780.588	4.1	PE(22:4(7Z,10Z,13Z,16Z)/P-18:0)	0.007	0.727	0.234	0.838	0.001	0.399	2892921	138562	2103230	183109	1763515	195442	838480	214866
782.567	4.2	[PC (16:0/20:4)] 1-hexadecanoyl-2-(5Z,8Z,11Z,14Z-eicosatetraenoyl)-sn-glycero-3-phosphocholine	0.017	1.385	0.131	0.841	0.615	0.947	59039125	2599935	81770434	6624135	68749636	4039639	77473071	4932306
784.509	3.9	[PS (18:2/18:2)] 1,2-di-(9Z,12Z-octadecadienoyl)-sn-glycero-3-phosphoserine	0.000	1.690	0.000	0.295	0.000	0.502	207845	16893	351205	17711	103616	20705	176414	10164
784.584	4.2	PC(18:2(9Z,12Z)/18:1(9Z))	0.000	4.581	0.044	0.765	0.470	0.920	10837590	476431	49645370	4092311	37998126	2792824	45686920	3302335
786.162	11.4	FAD	0.006	5.497	0.686	0.901	0.110	1.375	11104	2677	61040	11160	55022	9158	83949	6049
786.528	3.9	[PS (18:1/18:2)] 1-(9Z-octadecenoyl)-2-(9Z,12Z-octadecadienoyl)-sn-glycero-3-phosphoserine	0.009	3.595	0.250	0.587	0.022	0.410	66617	18241	239493	43660	140487	67222	98246	12673
786.599	4.2	[PC (18:1/18:1)] 1-(9Z-octadecenoyl)-2-(9Z-octadecenoyl)-sn-glycero-3-phosphocholine	0.000	8.411	0.001	0.525	0.001	0.675	18885418	933003	158843698	8918327	83346312	12740651	107206989	3373746
787.667	7.2	SM(d18:1/22:0)	0.044	0.251	0.040	0.387	0.015	0.228	411384	115433	103120	22330	39894	13064	23549	5113
787.668	4.3	SM(d18:1/22:0)	0.000	0.121	0.276	0.441	0.126	0.202	1944618	156152	234341	101680	103378	43506	47281	15037
788.542	3.9	[PS (18:1/18:1)] 1,2-di-(9E-octadecenoyl)-sn-glycero-3-phosphoserine	0.000	3.552	0.000	0.197	0.000	0.223	1529240	127629	5431788	152452	1072626	130528	1213765	88231
788.615	4.2	[PC (18:0/18:1)] 1-octadecanoyl-2-(9Z-octadecenoyl)-sn-glycero-3-phosphocholine	0.002	2.812	0.737	0.933	0.024	0.618	17543304	993575	49338569	5375315	46025999	7889333	30485282	4560488

788.615	11.2	[PC (18:0/18:1)] 1-octadecanoyl-2-(9Z-octadecenoyl)-sn-glycero-3-phosphocholine	0.004	6.813	0.189	1.825	0.057	1.822	2688	172	18313	3076	33424	9723	33364	5938
789.545	3.9	[PG (8:0/8:0)] 1-(8-[5]-ladderane-octanyl)-2-(8-[3]-ladderane-octanyl)-sn-glycero-3-phospho-(1'-sn-glycerol)	0.000	3.458	0.000	0.193	0.000	0.222	729060	67819	2520972	70836	486982	68645	560633	41867
790.557	3.9	PS(18:0/18:1(9Z))	0.000	2.024	0.000	0.208	0.000	0.179	7595947	489407	15371330	388631	3204341	423004	2756318	360660
790.574	4.1	PC(20:5(5Z,8Z,11Z,14Z,17Z)/P-18:1(11Z))	0.027	0.496	0.867	1.055	0.404	0.706	2283162	318565	1131687	310012	1193942	182862	798611	219861
791.560	5.7	[PG (8:0/8:0)] 1-(8-[3]-ladderane-octanyl)-2-(8-[3]-ladderane-octanyl)-sn-glycero-3-phospho-(1'-sn-glycerol)	0.038	4.692	0.333	0.587	0.047	0.265	15234	3208	71477	20131	41955	20895	18954	5356
791.561	3.9	[PG (8:0/8:0)] 1-(8-[3]-ladderane-octanyl)-2-(8-[3]-ladderane-octanyl)-sn-glycero-3-phospho-(1'-sn-glycerol)	0.000	1.978	0.000	0.214	0.000	0.188	3578240	217192	7076786	163475	1515528	198436	1329047	165168
792.551	4.1	[PE (18:0/22:6)] 1-octadecanoyl-2-(4Z,7Z,10Z,13Z,16Z,19Z-docosahexaenoyl)-sn-glycero-3-phosphoethanolamine	0.004	3.283	0.016	0.523	0.015	0.517	2271236	113028	7455627	1022603	3897360	394564	3853831	304102
792.572	3.8	[PS (16:0/20:0)] 1-hexadecanoyl-2-eicosanoyl-sn-glycero-3-phosphoserine	0.027	6.984	0.793	0.914	0.763	1.103	44122	10953	308145	85896	281511	47567	340010	55382
794.568	4.1	PC(15:0/22:5(4Z,7Z,10Z,13Z,16Z))	0.000	42.086	0.000	0.421	0.000	0.447	239032	78848	10059852	557896	4238280	349217	4494654	292291
794.604	4.1	[PC (18:1/20:4)] 1-(1Z-octadecenyl)-2-(5Z,8Z,11Z,14Z-eicosatetraenoyl)-sn-glycero-3-phosphocholine	0.000	0.532	0.035	0.746	0.007	0.648	49417600	2428662	26300588	2225281	19608442	1515630	17044472	1220234
796.584	4.1	PE(18:0/22:4(7Z,10Z,13Z,16Z))	0.000	57.557	0.011	0.726	0.015	0.746	150879	22040	8684186	591323	6303598	466119	6480350	441904
798.600	4.2	PE(18:1(11Z)/22:2(13Z,16Z))	0.000	122.178	0.004	0.653	0.023	0.750	35224	14441	4303626	324674	2808611	221082	3226995	218752
800.615	4.1	[PE (20:0/20:2)] 1-eicosanoyl-2-(11Z,14Z-eicosadienoyl)-sn-glycero-3-phosphoethanolamine	0.000	26.034	0.010	0.697	0.030	0.762	226341	64772	5892605	415004	4104655	384019	4489112	366659
801.564	3.8	PG(18:0/20:3(5Z,8Z,11Z))	0.007	37.551	0.013	0.175	0.013	0.167	4444	1796	166865	37132	29244	6917	27926	3565
803.520	3.7	[PG (8:0/8:0)] 1-(8-[5]-ladderane-octanoyl)-2-(8-[3]-ladderane-octanyl)-sn-glycero-3-phospho-(1'-sn-glycerol)	0.001	6.368	0.001	0.267	0.003	0.377	366444	24920	2333657	278167	623814	52614	879136	56388
804.573	3.8	[GP (18:0/18:0)] 1-octadecanoyl-2-(9Z-octadecenoyl)-sn-glycero-3-phosphothreonine	0.001	2.679	0.000	0.191	0.000	0.109	58966	9835	157964	16936	30112	7766	17184	3689
805.536	3.7	[PG (8:0/8:0)] 1-(8-[3]-ladderane-octanoyl)-2-(8-[3]-ladderane-octanyl)-sn-glycero-3-phospho-(1'-sn-glycerol)	0.001	5.060	0.001	0.258	0.003	0.402	213361	18409	1079596	121640	278838	45222	433645	32301
806.567	4.1	[PC (16:0/22:6)] 1-hexadecanoyl-2-(4Z,7Z,10Z,13Z,16Z,19Z-docosahexaenoyl)-sn-glycero-3-phosphocholine	0.001	2.356	0.013	0.685	0.235	0.864	13532411	642079	31888238	2712667	21846582	1586205	27556831	2063775
808.511	3.9	PS(20:3(8Z,11Z,14Z)/18:3(9Z,12Z,15Z))	0.002	9.859	0.764	1.077	0.002	1.805	19084	3676	188140	29630	202603	36184	339548	17962
808.583	4.1	[PC (18:1/20:4)] 1-(9Z-octadecenoyl)-2-(5Z,8Z,11Z,14Z-eicosatetraenoyl)-sn-glycero-3-phosphocholine	0.001	2.184	0.022	0.720	0.327	0.889	32952015	1565037	71979679	6098641	51791625	3723364	64009299	4705802
810.131	12.3	Acetyl-CoA	0.004	4.363	0.480	1.213	0.000	2.257	7449	1664	32503	5394	39430	7700	73358	4609
810.526	3.8	1-20:2-2-18:3-phosphatidylserine	0.000	3.432	0.000	0.186	0.000	0.291	338941	30936	1163339	68173	216060	30770	338217	42485
812.541	3.8	[PS (18:0/20:4)] 1-octadecanoyl-2-(5Z,8Z,11Z,14Z-eicosatetraenoyl)-sn-glycero-3-phosphoserine	0.001	1.336	0.000	0.162	0.000	0.197	6785006	340096	9066618	292920	1467731	128029	1782360	108356

812.615	4.1	PC(16:1(9Z)/22:2(13Z,16Z))	0.000	#DIV/0!	0.156	0.840	0.086	0.821	0	0	23435443	1668250	19693670	1774178	19245841	1429895
814.630	4.1	[PC (18:0/20:2)] 1-octadecanoyl-2-(11Z,14Z-eicosadienoyl)-sn-glycero-3-phosphocholine	0.000	37.217	0.087	0.795	0.146	0.850	243492	76459	9061968	579093	7206503	778447	7700869	639529
816.572	3.8	1-20:0-2-18:2-phosphatidylserine	0.000	5.066	0.000	0.138	0.000	0.178	170555	23618	864055	29429	118840	31070	154101	39185
818.566	4.1	PE(20:1(11Z)/22:6(4Z,7Z,10Z,13Z,16Z,19Z))	0.033	5.115	0.153	0.529	0.709	1.117	22600	3185	115596	31781	61127	9648	129164	14249
818.590	3.8	1-20:0-2-18:1-phosphatidylserine	0.001	3.346	0.000	0.175	0.000	0.211	88782	18211	297094	33727	51889	16717	62541	12479
820.584	4.1	PE(20:0/22:6(4Z,7Z,10Z,13Z,16Z,19Z))	0.000	65.367	0.017	0.548	0.392	0.872	30061	12174	1965015	202963	1077523	231574	1714167	193200
820.619	4.1	PC(22:4(7Z,10Z,13Z,16Z)/P-18:1(11Z))	0.034	1.269	0.002	0.618	0.001	0.572	6052148	322329	7681141	552192	4746417	392369	4393633	317952
821.530	3.8	PG(18:1(11Z)/22:6(4Z,7Z,10Z,13Z,16Z,19Z))	0.000	8.558	0.000	0.126	0.000	0.163	488042	37702	4176819	404702	526630	45219	679558	53415
823.547	3.7	[PG (18:0/22:6)] 1-octadecanoyl-2-(4Z,7Z,10Z,13Z,16Z,19Z-docosahexaenoyl)-sn-glycero-3-phospho-(1'-sn-glycerol)	0.000	7.487	0.000	0.130	0.000	0.156	273416	16706	2047161	200983	266581	24103	319952	26868
824.555	4.3	[SP] (3'-sulfo)Galbeta-Cer(d18:0/2-OH-18:0)	0.020	10.874	0.029	0.193	0.032	0.212	17433	4771	189570	50952	36554	9525	40225	7315
824.651	4.1	PC(22:2(13Z,16Z)/P-18:1(11Z))	0.001	0.425	0.135	1.857	0.221	1.593	1160768	80087	493376	102246	916255	228394	785866	193747
826.631	4.1	PE(18:2(9Z,12Z)/24:1(15Z))	0.000	14.486	0.383	1.332	0.847	0.959	13199	3972	191213	22866	254745	63735	183350	32247
828.255	17.4	Deshydroxy-C-1027 chromophore	0.000	6.077	0.407	0.895	0.164	1.203	692980	42001	4211460	267146	3769158	430367	5065584	487695
832.582	4.1	[PC (18:1/22:6)] 1-(11Z-octadecenoyl)-2-(4Z,7Z,10Z,13Z,16Z,19Z-docosahexaenoyl)-sn-glycero-3-phosphocholine	0.000	2.810	0.002	0.545	0.081	0.794	6291022	371516	17676156	1513805	9636543	719069	14038115	1066821
832.588	17.4	[PC (18:1/22:6)] 1-(11Z-octadecenoyl)-2-(4Z,7Z,10Z,13Z,16Z,19Z-docosahexaenoyl)-sn-glycero-3-phosphocholine	0.000	7.079	0.067	3.913	0.014	6.790	2645	1470	18724	2394	73272	23434	127135	29417
834.525	3.8	PS(18:1(9Z)/22:6(4Z,7Z,10Z,13Z,16Z,19Z))	0.000	1.497	0.000	0.253	0.000	0.377	579990	27315	868089	31947	219721	15239	327305	24290
834.599	4.1	[PC (18:1/22:5)] 1-(11Z-octadecenoyl)-2-(7Z,10Z,13Z,16Z,19Z-docosapentaenoyl)-sn-glycero-3-phosphocholine	0.000	3.012	0.006	0.655	0.129	0.832	9907410	522500	29840641	2377923	19557957	1535773	24819354	1866166
836.541	5.4	[PS (18:0/22:6)] 1-octadecanoyl-2-(4Z,7Z,10Z,13Z,16Z,19Z-docosahexaenoyl)-sn-glycero-3-phosphoserine	0.043	4.584	0.151	0.499	0.298	0.653	8812	2198	40391	11761	20149	3465	26369	3550
836.542	3.8	[PS (18:0/22:6)] 1-octadecanoyl-2-(4Z,7Z,10Z,13Z,16Z,19Z-docosahexaenoyl)-sn-glycero-3-phosphoserine	0.000	3.270	0.000	0.265	0.000	0.321	4272276	218613	13969940	686775	3703315	329537	4479370	279062
836.615	4.1	[PC (18:0/22:5)] 1-octadecanoyl-2-(4Z,7Z,10Z,13Z,16Z-docosapentaenoyl)-sn-glycero-3-phosphocholine	0.000	2.604	0.005	0.644	0.024	0.738	9534439	482168	24825470	1985124	15991494	1319726	18332575	1311154
838.557	3.8	PS(18:0/22:5(7Z,10Z,13Z,16Z,19Z))	0.000	3.205	0.000	0.187	0.000	0.218	4754168	278631	15235261	822768	2841561	276339	3314066	222007
838.630	4.1	[PC (18:0/22:4)] 1-octadecanoyl-2-(7Z,10Z,13Z,16Z-docosatetraenoyl)-sn-glycero-3-phosphocholine	0.000	2.243	0.012	0.714	0.026	0.768	3663831	227909	8217194	533885	5865092	555584	6307736	498195
840.573	3.8	1-22:1-2-18:3-phosphatidylserine	0.000	2.316	0.000	0.201	0.000	0.217	2260421	141206	5234182	260090	1052231	101401	1136606	84393

842.661	4.2	[PC (20:0/20:2)] 1-eicosanoyl-2-(11Z,14Z-eicosadienoyl)-sn-glycero-3-phosphocholine	0.003	37.410	0.172	2.751	0.034	2.561	1834	1250	68601	12505	188726	75062	175655	37314
848.613	4.2	PE(20:5(5Z,8Z,11Z,14Z,17Z)/24:1(15Z))	0.003	13.524	0.008	0.301	0.008	0.276	19862	3422	268605	46695	80936	27201	74244	13199
854.567	4.1	[PC (20:5/22:5)] 1-(5Z,8Z,11Z,14Z,17Z-eicosapentaenoyl)-2-(7Z,10Z,13Z,16Z,19Z-docosapentaenoyl)-sn-glycero-3-phosphocholine	0.002	1.716	0.000	0.086	0.000	0.350	1595233	100093	2738074	222382	234965	76915	957568	46731
859.486	3.8	Avermectin B1b	0.000	17.007	0.000	0.077	0.000	0.090	17908	4870	304551	34078	23385	2836	27395	7850
863.562	3.8	[PI (18:0/18:0)] 1,2-di-(9Z-octadecenoyl)-sn-glycero-3-phospho-(1'-myo-inositol)	0.013	1.382	0.000	0.491	0.001	0.544	763978	48789	1056137	79108	518102	57194	574102	59584
866.661	4.1	PC(18:3(6Z,9Z,12Z)/24:1(15Z))	0.018	3.088	0.999	1.001	0.846	0.948	98447	24026	303998	60906	304155	90244	288236	50589
867.593	3.8	[PI (18:0/18:0)] 1,2-dioctadecanoyl-sn-glycero-3-phospho-(1'-myo-inositol)	0.000	4.913	0.000	0.061	0.000	0.082	87989	17467	432262	35130	26183	7113	35653	12530
868.606	3.8	1-24:1-2-18:3-phosphatidylserine	0.003	3.107	0.001	0.050	0.001	0.049	77993	9534	242360	31766	12019	2903	11884	1661
878.568	4.1	[PC (22:6/22:6)] 1,2-di-(4Z,7Z,10Z,13Z,16Z,19Z-docosahexaenoyl)-sn-glycero-3-phosphocholine	0.007	5.570	0.006	0.124	0.007	0.206	47952	6353	267105	50853	33161	6046	54993	16801
885.546	3.8	PI(16:0/22:5(4Z,7Z,10Z,13Z,16Z))	0.000	3.381	0.000	0.145	0.000	0.200	1522687	81525	5148783	470315	746632	57639	1030836	60365
887.562	3.8	[PI (18:0/20:4)] 1-octadecanoyl-2-(5Z,8Z,11Z,14Z-eicosatetraenoyl)-sn-glycero-3-phospho-(1'-myo-inositol)	0.000	2.398	0.000	0.166	0.000	0.179	3678084	228236	8818608	530396	1462597	147251	1577097	117719
888.647	13.6	PC(22:1(13Z)/22:6(4Z,7Z,10Z,13Z,16Z,19Z))	0.042	2.262	0.689	1.193	0.737	1.085	8344	3386	18871	2968	22520	8168	20471	3539
890.659	4.1	Lactosylceramide(d18	0.002	#DIV/0!	0.023	0.411	0.816	1.062	0	0	35853	6014	14730	5060	38076	7075
891.512	3.7	Avermectin A2b	0.000	1.668	0.000	0.063	0.000	0.130	959367	50663	1599805	93913	100674	19120	207639	18746
892.677	4.1	PC(20:4(5Z,8Z,11Z,14Z)/24:1(15Z))	0.013	38.352	0.459	0.767	0.038	2.457	1648	1647	63190	16396	48491	9374	155280	32682
908.532	3.9	7-hydroxy-chlorophyll a	0.001	3.642	0.001	0.197	0.002	0.339	41559	8237	151346	19014	29849	4896	51380	6847
988.763	19.3	Lactosylceramide(d18	0.012	4.343	0.474	1.430	0.084	2.511	77380	8305	336069	68139	480509	177507	843990	234490
613.1384	17.4	CMP-N-acetylneuraminate	#DIV/0!	#DIV/0!	0.008	#DIV/0!	0.006	#DIV/0!	0	0	0	0	87461	20597	73352	16025
611.1449	17.4	Glutathione disulfide	0.012	70.861	0.008	78.553	0.007	71.932	224	143	15874	4067	1382853	321325	1141846	252763
606.0744	15.0	UDP-N-acetyl-D-glucosamine	0.107	3.312	0.008	33.266	0.016	46.857	1890	501	6260	2216	228890	52083	293334	80379
565.0477	16.1	UDP-glucose	0.897	1.058	0.002	24.657	0.007	50.760	3004	1026	3178	816	84678	13236	161324	36510
540.0537	14.2	Cyclic ADP-ribose	0.003	2.527	0.006	2.457	0.002	3.342	81280	2983	205394	23267	517821	72865	686404	85933
522.9671	14.6	XTP	0.000	0.514	0.555	1.312	0.968	0.983	41901	1935	21536	1473	27568	9444	21166	8793
519.0717	14.6	Pseudohypericin	0.178	#DIV/0!	0.010	95.910	0.005	211.802	0	0	889	567	102276	25452	188216	38313
506.9838	12.9	2'-azido-dCTP	0.005	6.294	0.001	32.000	0.000	47.704	166	105	1045	199	32627	4852	49829	4501
466.1233	14.7	Cys-Trp-Cys-Gly	0.002	2.444	0.411	0.997	0.512	0.877	30213	1798	73836	7785	81258	3338	64729	10846
451.1262	16.0	[Fv hydroxy,trihydroxy(3:0/9:1)] 3-(3,4-Dihydroxyphenyl)-1-(3-beta-D-glucopyranosyl-2,4,6-trihydroxyphenyl)-1-propanone	0.526	0.789	0.052	0.204	0.470	0.723	10990	2049	8674	2855	1461	631	6271	1328
445.053	16.3	CDP-ethanolamine	0.017	90.870	0.051	2.263	0.012	6.306	71	71	6418	1794	14384	2988	40471	9037
426.0223	16.6	ADP	0.001	5.765	0.006	3.757	0.007	7.457	7109	1648	40984	5053	161749	26768	305631	61263

426.0222	14.2	ADP	0.001	2.030	0.067	1.385	0.006	1.743	45366	3667	92094	7201	130956	16330	160524	16074
421.1035	16.0	Ala-Asp-Asp-Cys	0.002	4.234	0.001	43.095	0.000	53.933	323	150	1366	195	55959	7164	73660	7933
395.0078	14.2	Sucralose	0.000	2.867	0.689	1.083	0.000	0.622	16497	2085	47294	1097	51411	9640	29413	2284
394.0042	14.3	Molybdopterin	0.000	2.297	0.000	1.588	0.000	1.468	200452	7979	460367	10911	730603	26244	675887	23017
386.0171	14.2	dCDP	0.003	79.854	0.002	5.415	0.002	5.213	143	91	11423	2116	64715	9329	59554	8886
383.1196	18.3	Acetyl-maltose	0.003	7.448	0.001	8.864	0.001	15.411	960	87	7151	1192	71815	9073	110209	16295
381.1099	14.6	Cys-Ser-Ser-Ser	0.001	20.367	0.004	0.381	0.002	0.267	14706	2233	299516	39921	118273	19079	79873	13745
373.0508	15.1	Glucocochlearin	0.002	0.370	0.477	1.128	0.020	0.604	27066	3026	10008	682	11244	1497	6047	1181
362.8913	14.3	Tetrachlorvinphos	0.001	0.139	0.996	1.002	0.492	1.222	32442	4499	4519	682	4514	1044	5522	1212
361.0073	14.6	Coumaphos	0.000	3.616	0.033	1.641	0.010	4.258	5051	1234	18264	1872	31499	4554	77775	14733
357.077	15.1	Miraxanthin-I	0.034	0.587	0.025	1.379	0.025	1.425	509822	71961	299123	37827	419346	21513	426148	29338
347.1031	12.9	Camptothecin	0.318	2.407	0.025	7.015	0.004	27.130	2478	1365	5965	2947	50209	14060	161819	31407
346.0558	16.6	AMP	0.006	4.043	0.002	3.539	0.002	6.331	164605	21324	665524	113715	2461240	334887	4213754	643282
346.0558	14.2	AMP	0.006	1.869	0.171	1.211	0.021	1.480	58250	1833	108853	11104	135239	13938	161079	15231
341.1088	15.2	Sucrose	0.083	1.437	0.113	1.543	0.045	1.734	35140	5374	50509	5869	81287	15709	87575	13857
341.1088	14.5	Sucrose	0.004	2.684	0.001	0.217	0.002	0.263	20618	2078	55340	7212	12263	3233	14552	6025
338.0488	17.3	S-sulfanylglutathione	0.080	#DIV/0!	0.006	326.271	0.005	320.140	0	0	250	114	98076	21473	80194	16914
334.1257	16.0	N4-(Acetyl-beta-D-glucosaminyl)asparagine	0.000	2.554	0.000	0.061	0.000	0.112	52094	4720	133053	5745	8093	1606	14937	2720
334.0334	15.1	Nicotinate D-ribonucleotide	0.374	0.780	0.276	1.213	0.763	1.088	35406	6309	27626	5479	35437	3923	30062	5634
333.0594	16.0	sn-glycero-3-Phospho-1-inositol	0.000	8.156	0.002	0.492	0.140	0.806	191790	5941	1564278	149856	794747	73388	1261008	113815
330.0544	14.2	Piroxicam	0.001	27.765	0.000	14.139	0.000	17.261	1757	435	48797	6880	715711	41686	842295	41319
330.0544	17.4	Piroxicam	0.042	#DIV/0!	0.000	48.593	0.000	50.512	0	0	2419	890	134011	14740	122171	14486
328.0454	16.6	3',5'-Cyclic AMP	0.006	5.171	0.004	3.765	0.002	6.333	15848	2617	81951	14808	326998	50244	519028	77479
328.0453	14.2	3',5'-Cyclic AMP	0.003	2.442	0.010	1.529	0.002	1.777	68013	3037	166079	18373	262217	23416	295102	25274
325.1252	15.0	2,2,4-Trimethyl-3-(4-fluorophenyl)-2H-1-benzopyran-7-ol acetate	0.001	0.189	0.000	4.700	0.000	5.907	32171	4131	6094	1892	29950	2803	35999	2571
323.1065	12.8	Acetohexamide	0.432	0.801	0.568	1.063	0.657	1.131	40950	5474	32811	8227	26758	6082	37112	4340
323.0289	15.1	UMP	0.058	1.894	0.005	30.054	0.010	35.383	958	290	1813	275	58587	11791	64161	15455
323.0286	16.2	UMP	0.958	0.978	0.001	12.168	0.004	20.680	20309	6341	19866	5093	265025	34338	410826	76990
323.0286	17.8	UMP	0.446	1.360	0.001	7.950	0.002	18.426	6459	1810	8783	2292	75724	10319	161841	26763
323.0238	10.0	Chlorobenzilate	0.591	0.823	0.001	2.128	0.000	3.830	453313	127034	373182	65075	820126	74232	1429458	115442
323.0234	15.1	Chlorobenzilate	0.003	0.474	0.063	0.610	0.093	0.528	37161	3938	17625	2770	10432	1964	9308	3496
322.0446	15.2	CMP	0.004	2.461	0.081	1.446	0.019	2.415	28362	1896	69803	8408	104602	15171	168568	29636
322.0446	16.0	CMP	0.002	1.795	0.001	2.107	0.000	2.095	38040	1853	68269	5579	146186	13337	143038	10185
321.0726	11.6	3-hydroxy-2-oxindole-3-acetyl-asp	0.890	1.045	0.540	1.084	0.036	1.732	27410	5167	28633	6893	33668	3783	49601	5090

317.0556	17.1	Prekinamycin	0.519	1.213	0.000	13.355	0.001	11.365	2046	473	2482	449	36527	2714	28207	3496
308.0988	13.2	N-Acetylneuraminate	0.018	3.057	0.007	0.117	0.023	0.370	17209	2886	52609	10390	6507	1192	19484	4693
307.1147	15.7	S-8-methylthiooctylhydroximoyl-L-cysteine	0.000	3.924	0.793	1.033	0.346	1.100	24963	1236	97954	6069	101253	10569	107795	7844
307.0823	14.5	Allamandin	0.175	#DIV/0!	0.228	220.425	0.002	87.625	0	0	1721	1088	455125	330013	150772	25771
306.0767	15.8	Glutathione	0.013	3.862	0.000	4.851	0.000	4.327	15812	2570	61070	12107	262144	25864	264273	25289
306.0766	17.4	Glutathione	0.009	13.124	0.004	70.176	0.003	61.423	1353	221	17751	3972	1289277	248443	1090333	204657
306.0766	14.2	Glutathione	0.007	15.201	0.002	127.197	0.001	209.107	8087	1262	122937	25843	16762955	2965101	25706999	3517712
306.0766	14.8	Glutathione	0.006	136.196	0.026	4.184	0.014	6.550	351	237	47800	10218	213036	53031	313082	71728
305.0181	15.0	2',3'-Cyclic UMP	0.044	0.475	0.001	48.797	0.003	54.911	1891	362	899	195	48639	6016	49356	8885
304.0707	14.8	Phenylamil	0.000	0.228	0.015	2.578	0.009	2.448	90937	5765	20709	2935	46966	7417	50700	7524
304.0608	17.1	Atherospermidine	0.098	5.162	0.005	26.663	0.004	24.346	934	560	4823	1894	144230	29857	117415	22930
302.9988	14.5	2-bromoporphobilinogen	#DIV/0!	#DIV/0!	0.022	#DIV/0!	0.007	#DIV/0!	0	0	0	0	35062	10728	44871	10059
300.0489	15.0	N-Acetyl-D-glucosamine 6-phosphate	0.003	0.428	0.000	27.336	0.000	26.704	9399	1061	4026	741	113401	10901	107504	9864
294.8584	17.8	Cu:pyrimidine-2,6-bis(thiocarboxylate)	0.000	0.529	0.000	1.451	0.000	1.457	1145474	19390	606055	16528	891566	22122	883012	21022
294.0543	15.0	Acetylsulfamethoxazole	0.019	3.509	0.159	1.309	0.049	1.550	9833	3804	34503	7312	47175	3323	53480	3050
291.0835	15.9	EDTA	0.915	0.985	0.192	1.218	0.007	1.440	257817	21274	254070	26769	309099	28751	365788	16752
289.0331	16.1	Sedoheptulose 7-phosphate	0.012	667.439	0.112	0.450	0.453	1.314	86	86	57173	14738	27961	6023	75113	17550
282.0844	12.7	Guanosine	0.092	2.694	0.007	17.598	0.001	27.553	1991	544	5366	1600	105504	22447	147836	20218
282.0384	15.0	N2-Acetyl-L-aminoadipyl-delta-phosphate	0.001	0.551	0.000	9.289	0.000	7.385	344262	22871	189623	7957	1769531	35912	1400269	76028
281.0867	15.2	Xylobiose	0.206	1.802	0.001	2.933	0.000	3.919	7782	2625	14027	3746	44662	4656	54971	3389
277.0587	14.7	Pyrimidine 5'-deoxynucleotide	0.000	3.418	0.283	0.859	0.015	0.730	99414	9335	339817	12208	288969	41302	247995	25880
276.0158	14.9	N'-Phosphoguanidinoethyl methyl phosphate	0.000	#DIV/0!	0.001	3.125	0.000	3.532	0	0	27635	1494	84262	8662	97609	4915
274.003	14.8	dihydrodopachrome sulfate	0.003	2.920	0.633	1.155	0.556	0.885	12062	2179	35226	4741	38061	3222	31190	4623
273.0382	15.1	1-Deoxy-D-altro-heptulose 7-phosphate	0.036	57.932	0.023	2.909	0.004	8.893	298	230	17243	5968	55238	11813	153336	27243
273.0081	14.9	ferulic acid sulfate or isomer	0.000	1.497	0.014	0.919	0.002	0.793	162337	6893	242992	5476	223156	1214	192788	9624
266.0895	11.2	Adenosine	0.164	2.320	0.000	62.644	0.000	97.751	236	115	548	170	41172	4400	53539	5085
261.038	16.0	Sorbitol 6-phosphate	0.000	10.888	0.370	0.874	0.042	0.751	5629	467	61289	4950	54729	4921	46001	4303
260.0888	17.4	Ala-Asp-Gly	0.092	#DIV/0!	0.001	96.105	0.008	88.430	0	0	268	129	30919	4081	23708	5527
260.0887	14.2	Ala-Asp-Gly	0.363	#DIV/0!	0.002	860.877	0.000	1265.632	0	0	70	70	72278	11516	88551	10067
260.0887	15.8	Ala-Asp-Gly	0.014	3.729	0.005	3.303	0.491	1.212	6143	553	22908	4567	65343	9735	27766	5037
259.0224	16.0	D-Glucose 6-phosphate	0.000	15.450	0.872	0.950	0.938	0.989	18839	914	291063	31549	283649	31732	287779	25915
256.0954	14.6	sn-glycero-3-Phosphocholine	0.005	51.630	0.005	0.008	0.005	0.002	940	238	48523	10143	370	175	86	86
255.2328	16.6	Hexadecanoic acid	0.388	2.479	0.451	0.426	0.361	0.361	29281	4820	72599	45689	35172	4528	26189	8749
255.028	8.3	validone-7-phosphate	#DIV/0!	#DIV/0!	0.002	#DIV/0!	0.009	#DIV/0!	0	0	0	0	1274	226	4248	1033

253.0593	8.2	N-δ-(phosphonoacetyl)-L-ornithine	0.071	0.169	0.892	1.069	0.073	0.163	61287	22275	10348	3789	9248	6856	1688	1302
251.0784	9.6	Deoxyinosine	0.072	2.383	0.007	21.680	0.000	35.912	427	214	1017	200	23962	5198	36509	3636
249.0491	10.2	[FA (10:0)] 10-bromo-decanoic acid	0.829	0.958	0.623	1.046	0.181	1.255	540187	63828	517742	78192	561942	35460	649837	44887
249.0219	14.8	4,4'-Sulfonyldiphenol	0.016	93.496	0.299	0.581	0.770	0.890	3205	569	299664	83197	190574	52832	266653	71702
246.9263	14.8	4-iodobenzoate	0.005	2.190	0.220	0.857	0.036	0.648	27367	2977	59944	7259	49492	2424	38847	4095
246.0463	12.6	DCI	0.216	2.182	0.187	1.549	0.006	4.385	4633	1160	10112	3784	17456	3540	44345	8112
245.1253	14.3	N2-(D-1-Carboxyethyl)-L-arginine	0.003	#DIV/0!	0.009	3.588	0.022	4.363	0	0	5346	980	20320	3766	23326	5536
245.0429	12.6	Glycerophosphoglycerol	0.161	1.875	0.230	1.382	0.007	3.793	110540	16145	207248	57860	309650	55373	786174	137622
243.062	12.0	Uridine	0.624	1.141	0.000	3.804	0.000	4.458	31264	5187	35668	6952	150479	11204	159005	13355
242.0779	17.4	Cytidine	0.015	#DIV/0!	0.000	47.074	0.000	38.864	0	0	1753	480	84619	9374	68121	7938
242.0779	14.5	Cytidine	0.173	#DIV/0!	0.025	3.304	0.002	5.032	0	0	102246	64435	405375	92375	514525	73376
241.0117	16.2	D-myo-Inositol 1,2-cyclic phosphate	0.565	1.086	0.000	7.483	0.000	8.309	471460	37026	511877	56509	3940157	391127	4253205	369960
239.0163	16.1	L-Cystine	0.141	0.590	0.012	0.200	0.015	0.259	181383	39734	107102	21874	22647	2238	27763	2666
238.8915	16.6	Trimetaphosphate	0.002	4.263	0.009	3.242	0.007	4.750	1715	301	7314	1040	24192	4223	34742	6368
237.0913	10.4	HEPES	0.416	2.612	0.373	0.283	0.304	4.428	1026	348	2679	1842	853	396	11865	7896
229.0114	15.7	D-Ribose 5-phosphate	0.000	33.235	0.003	0.424	0.063	0.718	1642	283	54571	6274	24159	3778	39200	3151
228.0986	17.4	Asn-Pro	0.015	18.730	0.000	45.629	0.000	44.785	113	71	2112	549	105824	12739	94574	8480
228.0986	14.2	Asn-Pro	0.009	#DIV/0!	0.001	113.400	0.000	178.283	0	0	1553	377	188842	29439	276908	34500
225.0988	15.7	Carnosine	0.000	1.947	0.000	0.594	0.000	0.480	76753	8058	149469	4799	86409	8616	71729	9226
221.0662	18.3	6-Acetyl-D-glucose	0.000	6.593	0.004	5.612	0.001	8.207	1139	121	7510	597	42812	7145	61636	7045
220.1464	17.7	Procarbazine	0.003	3.785	0.003	0.187	0.002	0.159	103016	19537	389907	58559	69148	8847	61974	6177
220.1464	16.8	Procarbazine	0.091	0.589	0.008	3.706	0.010	3.266	85200	15787	50203	9091	186541	33024	163942	29069
220.1464	13.9	Procarbazine	0.954	1.012	0.000	0.389	0.000	0.258	280369	53717	283800	17383	108353	17180	73296	31186
220.1464	13.4	Procarbazine	0.142	1.553	0.179	1.617	0.015	0.441	61984	15953	96238	14387	163807	42208	42476	10649
220.1464	15.5	Procarbazine	0.015	0.531	0.235	1.430	0.589	0.904	122707	16218	65138	9314	81921	9475	58868	6170
217.0825	9.9	L-Ala-L-Glu	0.001	16.638	0.001	14.688	0.000	35.554	110	69	1828	263	26230	3900	64978	5943
216.0521	15.7	2-(Hydroxymethyl)-3-(acetamidomethylene)succinate	0.000	4.894	0.000	0.380	0.000	0.331	17602	677	86148	6266	33319	3277	28535	1393
215.0322	13.5	2-C-Methyl-D-erythritol 4-phosphate	0.689	1.125	0.503	0.792	0.075	1.551	51009	10214	57365	11557	46674	10117	88964	10877
215.0322	17.2	2-C-Methyl-D-erythritol 4-phosphate	0.137	1.537	0.036	1.368	0.002	1.883	15409	3152	23681	4002	35560	2607	44582	3047
215.0321	14.6	2-C-Methyl-D-erythritol 4-phosphate	0.000	0.182	0.762	1.168	0.950	0.978	406128	21739	73763	9094	83915	30648	72176	22858
214.048	15.7	sn-glycero-3-Phosphoethanolamine	0.000	4.422	0.000	0.505	0.000	0.518	1950659	62320	8625737	580139	4443028	375444	4464538	351885
213.0334	14.3	Clofibric acid	0.176	#DIV/0!	0.001	148.569	0.003	228.596	0	0	182	116	32472	4928	41636	7520
212.0572	16.6	N,N-Dihydroxy-L-tyrosine	0.005	2.824	0.027	1.664	0.633	1.143	8478	1592	23938	3466	39672	4885	27359	5963
211.0008	12.5	P-DPD	0.031	99.026	0.718	0.764	0.033	4.152	118	118	11660	3897	10006	2050	48416	12654

211.0008	17.8	P-DPD	0.026	4.715	0.007	3.792	0.001	7.303	1361	311	6417	1623	25013	4400	46859	5763
211.0008	16.6	P-DPD	0.002	4.007	0.002	3.018	0.001	4.746	32910	4875	131887	17627	412057	52714	625991	81210
211.0007	15.2	P-DPD	0.003	5.582	0.037	2.371	0.076	2.007	2966	525	16554	2590	40269	8433	33223	7380
211.0007	14.2	P-DPD	0.002	2.388	0.690	0.882	0.973	1.006	13519	1359	32286	3449	29414	6020	32470	4151
210.0878	17.4	Zalcitabine	0.009	8.892	0.001	31.341	0.001	28.574	916	277	8142	1782	269711	35891	232638	31187
210.0878	14.3	Zalcitabine	0.004	#DIV/0!	0.002	84.975	0.000	127.120	0	0	7264	1448	630431	101698	923458	111006
203.0279	14.4	4-Chloro-4'-biphenylol	0.000	1.810	0.003	0.248	0.009	0.212	43655	4897	79027	3669	19679	11798	16724	15334
203.0198	15.1	Oxaloglutarate	0.713	0.937	0.001	1.642	0.000	1.804	223660	30972	209460	20772	348780	4307	377820	13868
199.0717	14.2	3-(3,4-Dihydroxypyridin-1-yl)-L-alanine	0.049	#DIV/0!	0.001	64.749	0.001	108.009	0	0	479	185	25700	3318	51689	7665
198.0877	17.4	gamma-Glutamyl-beta-aminopropiononitrile	0.014	6.900	0.000	24.983	0.000	23.649	738	119	5094	1173	140195	16758	120474	13966
198.0876	14.2	gamma-Glutamyl-beta-aminopropiononitrile	0.005	4.478	0.001	41.263	0.001	63.841	304	63	1361	229	63714	8102	86857	12617
197.0561	17.3	(S)-2-Amino-3-(3-hydroxy-4-oxo-4H-pyridin-1-yl)propanoate	0.062	2.855	0.001	62.191	0.000	60.678	351	31	1001	272	67657	8538	60749	6696
197.056	14.2	(S)-2-Amino-3-(3-hydroxy-4-oxo-4H-pyridin-1-yl)propanoate	0.002	86.194	0.001	57.772	0.000	85.414	44	44	3819	627	233922	34442	326193	35944
195.0502	13.3	D-Gluconic acid	0.000	2.891	0.007	1.759	0.000	2.330	15011	1367	43399	3100	78116	8326	101127	8026
195.0502	14.0	D-Gluconic acid	0.328	0.651	0.000	3.008	0.015	4.361	49393	15576	32175	4104	101237	8438	140306	30022
194.9634	15.1	tetranitromethane	0.000	20.028	0.000	0.257	0.000	0.117	3206	816	64211	6324	15784	2952	7534	1535
194.0621	17.2	4-(2-Benzofuranyl)pyridine	0.565	1.241	0.000	20.808	0.000	21.077	2510	674	3115	760	70937	5616	65656	6702
193.0346	16.1	D-Glucuronate	0.637	1.127	0.000	3.997	0.000	4.670	13487	2040	15199	2857	60319	2453	70973	5698
192.99	16.6	2-Chloro-3-oxoadipate	0.013	6.062	0.003	3.269	0.001	5.061	3804	1373	23062	5228	76740	11016	116711	15494
191.0189	18.1	Citrate	0.000	9032.522	0.000	0.024	0.000	0.017	1085	453	9800562	926163	238036	46071	165082	35027
191.0011	15.2	furan dimethanol sulfate	0.388	1.332	0.000	2.765	0.000	3.059	9255	1526	12331	2989	35445	2335	37723	2241
189.0396	10.5	[FA hydroxy,oxo(7:0/2:0)] 4-hydroxy-2-oxo-Heptanedioic acid	0.027	2.267	0.177	0.672	0.940	1.015	15623	4243	35416	6191	25291	2550	35964	3390
189.004	15.2	Oxalosuccinate	0.134	1.528	0.001	2.158	0.000	2.518	29766	5487	45475	7812	102096	9485	114499	9694
187.0398	14.5	1-Hydroxy-2-naphthoate	0.010	#DIV/0!	0.011	72.027	0.002	90.206	0	0	494	122	37673	9362	44528	7274
185.0559	17.4	(S)-AMPA	0.009	4.130	0.000	28.714	0.001	25.672	415	97	1712	328	46143	5058	43959	5736
185.0558	14.2	(S)-AMPA	0.000	4.442	0.001	93.902	0.001	148.153	270	90	1201	117	119536	17551	177946	23512
183.9919	15.1	L-Serine O-sulfate	0.275	0.803	0.166	1.227	0.046	1.392	76766	9383	61622	9168	76967	3562	85754	1592
183.9919	17.1	L-Serine O-sulfate	0.188	1.761	0.000	8.351	0.000	7.288	4666	1425	8217	2041	66256	7376	59888	4539
183.9919	14.4	L-Serine O-sulfate	0.000	3.119	0.000	3.194	0.000	3.981	16141	1703	50349	3434	167854	9170	200458	7196
182.0742	13.9	Chlorphentermine	0.195	1.255	0.121	0.752	0.911	0.985	65052	7072	81620	9515	63053	4545	80393	4740
182.0619	17.3	p-Fluorophenylalanine	0.075	8.499	0.000	75.118	0.000	72.259	52	52	442	173	33319	2668	31968	2907
181.0709	13.9	D-Sorbitol	0.127	1.286	0.078	0.745	0.546	0.926	1185688	124178	1524288	159357	1165041	66540	1412126	77407
180.0657	13.0	L-Tyrosine	0.859	1.100	0.527	1.170	0.003	3.142	105461	43346	115978	37826	152067	40009	364394	48683
179.0552	14.7	D-Glucose	0.000	0.429	0.002	0.268	0.000	0.094	185815	10174	79641	5818	21749	11321	7501	4529

179.0552	17.2	D-Glucose	0.166	1.275	0.052	1.281	0.004	1.596	1946804	191904	2482615	297120	3280318	190592	3961288	250437
179.0453	17.4	Nicotinurate	0.000	4.707	0.001	37.572	0.001	33.623	986	323	4642	525	181283	25335	156087	21806
177.033	17.4	Cys-Gly	0.023	15.004	0.001	83.233	0.001	78.004	50	50	757	221	67285	9579	59051	7417
177.033	14.2	Cys-Gly	0.024	#DIV/0!	0.002	106.930	0.000	169.136	0	0	3053	959	347997	56320	516377	64076
176.935	15.9	Pyrophosphate	0.934	0.985	0.614	1.032	0.022	1.467	10555341	1194844	10395445	1464902	11332460	1034282	15249693	917019
176.0569	16.6	4-Hydroxy-4-methylglutamate	0.004	2.247	0.445	1.128	0.515	1.284	11233	1829	25244	3057	28650	2997	32415	9910
175.048	17.2	Allantoate	0.433	1.191	0.000	12.563	0.000	11.885	10054	894	11978	2127	150270	11307	142367	11006
175.048	14.2	Allantoate	0.001	3.700	0.004	3.195	0.001	4.806	4045	212	14966	1508	47859	6698	71935	9191
174.0874	16.1	L-Citrulline	0.000	35.759	0.000	0.203	0.000	0.268	3667	1071	131131	6792	26150	2554	35085	5020
173.0082	18.1	Phenylmethanesulfonyl fluoride	0.000	2144.115	0.000	0.017	0.000	0.013	174	122	373177	33054	6616	1439	4805	1334
171.0765	17.3	Glycylproline	0.439	1.291	0.000	28.028	0.000	28.205	1995	400	2575	593	77481	7929	72630	5525
171.0054	15.7	sn-Glycerol 3-phosphate	0.000	4.428	0.000	0.431	0.000	0.322	15089	738	66820	3698	29182	3165	21527	1175
171.0054	12.6	sn-Glycerol 3-phosphate	0.203	1.814	0.143	1.568	0.005	4.523	9080	1681	16476	4907	29449	6462	74518	12872
167.0567	15.5	N-Cyclopropylammelide	0.835	0.953	0.474	0.951	0.057	0.656	56456	11135	53820	4859	48229	5722	35301	6963
167.0567	16.6	N-Cyclopropylammelide	0.458	1.684	0.305	0.370	0.287	0.397	48870	5488	82299	41343	34829	5417	32668	6570
167.0566	13.2	N-Cyclopropylammelide	0.365	0.677	0.124	2.120	0.163	0.716	107125	33613	72495	11128	136057	33788	51942	7681
167.0566	13.9	N-Cyclopropylammelide	0.127	0.490	0.741	0.815	0.173	0.411	193484	47408	94847	34753	82230	11111	38973	8376
167.0566	12.1	N-Cyclopropylammelide	0.900	1.012	0.231	0.890	0.082	0.798	858420	45000	868987	68423	754396	57940	693076	59865
166.0976	17.4	2-Dimethylamino-5,6-dimethylpyrimidin-4-ol	0.363	#DIV/0!	0.001	205.838	0.000	207.200	0	0	243	243	60118	7700	50430	5669
166.0282	15.7	demethyl-phosphinothricin	0.000	3.431	0.000	0.505	0.000	0.519	163921	4799	562334	26736	288656	23011	292101	18869
165.0329	13.5	2-methylphosphinoyl-2-hydroxyacetate	0.251	1.317	0.000	3.296	0.001	5.355	36064	6994	47498	6226	163018	13399	254370	32647
164.0362	13.7	Formylanthranilate	#DIV/0!	#DIV/0!	0.177	#DIV/0!	#DIV/0!	#DIV/0!	0	0	0	0	94	60	0	0
161.0458	9.6	2-Dehydro-3-deoxy-L-rhamnonate	0.122	2.840	0.002	13.404	0.004	20.580	348	169	988	326	15886	2631	20325	3855
161.0096	14.2	4-Hydroxy-2-oxoglutarate	0.004	#DIV/0!	0.000	24.576	0.007	28.026	0	0	1681	339	42392	4946	47119	10428
157.0303	14.7	1,4-Naphthoquinone	0.003	0.139	0.076	2.820	0.029	2.736	19988	3284	2787	511	8883	2732	7627	1614
156.042	13.2	5-diazo-4-oxo-norvaline	0.637	0.895	0.277	1.389	0.020	0.559	52477	10628	46961	3183	62622	12613	26248	6217
149.046	11.6	D-Ribose	0.700	1.070	0.740	1.011	0.092	1.260	40096	5345	42901	4631	44731	2620	54058	3754
149.0447	8.7	D-Ribose	0.170	1.631	0.000	7.693	0.000	10.010	4855	958	7917	1781	65705	5592	79249	5471
146.064	15.1	5-methylthiopentanaldoxime	0.423	1.183	0.000	1.951	0.000	2.259	210449	31434	248877	33492	499791	17945	562323	14637
146.0577	15.1	L-Albizziine	0.684	1.095	0.000	1.852	0.000	2.203	23805	3427	26070	4181	53586	2474	57428	2482
144.0484	17.4	5-methylthiopentanonitrile oxide	0.042	8.837	0.000	85.150	0.000	79.448	51	51	451	148	40495	4916	35797	3643
144.0483	14.2	5-methylthiopentanonitrile oxide	0.175	#DIV/0!	0.003	803.583	0.000	776.295	0	0	141	89	68082	12668	109454	11501
135.03	10.3	[FA trihydroxy(4:0)] 2,3,4-trihydroxy-butanoic acid	0.645	1.234	0.000	3.710	0.073	1.678	4921	2044	6073	1272	22217	2289	10188	1597
135.03	9.6	[FA trihydroxy(4:0)] 2,3,4-trihydroxy-butanoic acid	0.737	1.152	0.000	9.204	0.000	15.693	1556	443	1792	518	17980	1413	28115	2275

134.046	17.4	4-Hydroxy-L-threonine	0.342	1.267	0.004	3.328	0.170	1.966	1127276	215045	1428307	211696	4322212	606786	2808540	850951
134.046	14.1	4-Hydroxy-L-threonine	0.000	1.712	0.001	0.703	0.003	0.769	2867209	86241	4908253	227052	3494855	144465	3772526	149356
134.046	16.6	4-Hydroxy-L-threonine	0.000	1.631	0.000	1.385	0.000	1.461	17527416	637819	28582051	1411383	39863493	514988	41744489	975396
134.046	15.2	4-Hydroxy-L-threonine	0.000	2.204	0.000	0.656	0.000	0.577	786282	28284	1732652	16618	1131579	25137	1000390	40237
133.0143	19.4	(S)-Malate	0.000	6.553	0.003	2.026	0.001	3.380	5645	943	36992	4024	73781	7698	125037	14686
132.0483	15.2	4-methylthiobutanaldoxime	0.147	1.427	0.001	2.254	0.001	2.586	26640	3907	38028	5978	88528	8519	98338	9752
132.0303	16.7	L-Aspartate	0.290	1.345	0.037	1.585	0.702	1.218	24380	5547	32797	5095	55952	7889	39958	17041
127.0194	14.5	[FA (9:1/3:0)] 2-nonene-4,6,8-triynal	0.002	0.094	0.005	6.927	0.001	8.855	183522	27949	17238	4519	122372	22478	152635	21223
126.0421	14.3	2-Hydroxy-4,6-diamino-1,3,5-triazine	0.005	#DIV/0!	0.008	14.180	0.003	22.455	0	0	647	135	10153	2229	14528	2517
